# Phage-first treatment results in subpopulation-driven collateral sensitivity to beta-lactams in an extensively drug-resistant *Pseudomonas aeruginosa*

**DOI:** 10.1128/aac.00048-26

**Published:** 2026-09-03

**Authors:** Claire Han, Karishma Patel, Haoran Gao, Jacob T. Sanborn, Thomas D. Nguyen, Jack F. Klem, Brian M. Ho, Patrick D. Kenney, Yanan Zhao, Troy D. Wood, Dwayne R. Roach, Liang Chen, Nicholas M. Smith

**Affiliations:** 1 Division of Clinical and Translational Therapeutics, School of Pharmacy and Pharmaceutical Sciences, University at Buffalo 15497 , Buffalo, New York, USA; 2 Department of Infectious Diseases, Jacobs School of Medicine and Biomedical Sciences, University at Buffalo 12291 , Buffalo, New York, USA; 3 Department of Chemistry, College of Arts and Sciences, University at Buffalo 429013 , Buffalo, New York, USA; 4 Department of Biology, San Diego State University 115105, San Diego, California, USA; Shionogi Inc., Florham Park, New Jersey, USA

**Keywords:** KPC, *Pseudomonas aeruginosa*, bacteriophage, beta-lactam, carbapenem, combination therapy, LPS, permeability, pharmacodynamics

## Abstract

Nosocomial *Pseudomonas aeruginosa* infections are among the most challenging infections to treat, and resistance to last-line agents, including polymyxins and aztreonam-based therapies, risks a future with limited or no clinical treatment options. Bacteriophages (phages) have emerged as a promising therapeutic option, both in cocktails and when given in combination with antibiotics. In this study, we show that the synergy between a lipopolysaccharide (LPS)-specific phage and antibiotics is driven by clinically relevant increases in beta-lactam permeability due to the selection of phage-resistant subpopulations. First, in the Hollow Fiber Infection Model (HFIM), we show that the combination of phage LUZ19 (pili-targeted), phage E215 (LPS-specific), and aztreonam (ATM) eradicated the laboratory host PAO1, whereas monotherapy with neither phage nor ATM could eradicate PAO1. Static time-kill studies (STKS) evaluated the LPS-specific phage PYO2 in combination with either aztreonam/avibactam (ATM/AVI) or polymyxin B (PMB) against an extensively drug-resistant clinical isolate of *P. aeruginosa* (AR-0231). PYO2 combined with ATM/AVI was determined to be synergistic, with a mean excess-over-bliss (EOB) of 0.350 (*P* < 0.05), while PYO2 with PMB was also synergistic, with a mean EOB of 0.361 (*P* < 0.05). Linear regression of STKS using sequential administration showed that, compared to antibiotic-first treatment, phage-first treatment reduced bacterial concentrations at 24 h by −6.19 log_10_ colony-forming unit (CFU)/mL (*P* < 0.05), with statistically significant interactions estimated for each antibiotic. These results indicated that bacterial pre-selection by PYO2 improved antibiotic activity. Mass spectrometry studies showed that the PYO2-resistant AR-0231 strain exhibited a >25% increase in the outer membrane permeability surface area coefficient for all major anti-*Pseudomonal* beta-lactams, including aztreonam. Altogether, our study provides mechanistic insights into phage-induced collateral sensitivity of beta-lactams. These results show how specific beta-lactams can be prioritized and optimized in phage-antibiotic combinations to maximize the antibacterial activity.

## INTRODUCTION

Antibiotic resistance remains a leading cause of treatment failure and recurrent infections worldwide (1–3). In addition to resistance, the persistence of bacterial cells in infection sites has emerged as a critical barrier to curing and preventing further resistance among these gram-negative ESKAPE “superbugs” (4, 5).

*Pseudomonas aeruginosa* is an ESKAPE pathogen that is associated with severe nosocomial infections, including pneumonia, surgical site infections, urinary tract infections, burn wound infections, and bloodstream infections (6, 7). According to the U.S. Centers for Disease Control and Prevention, multidrug-resistant *P. aeruginosa* was responsible for an estimated 32,600 infections and 2,700 deaths among hospitalized patients in the USA in 2019 (8), with a dwindling supply of available agents (9).

Anti-*Pseudomonal* beta-lactams are among the most clinically important agents when treating *P. aeruginosa infections* and include the following: carbapenems (e.g., meropenem [MEM] and imipenem [IPM]), aztreonam (ATM), ceftazidime (CAZ), cefepime (FEP), and piperacillin (PIP). Of these, ATM is especially important because it is the only beta-lactam stable against metallo-β-lactamases (MBLs). When combined with avibactam (AVI), which inhibits serine-β-lactamases, ATM-AVI retains activity against strains co-producing both resistance mechanisms (10, 11). ATM is also frequently used as an inhaled formulation among patients for anti-inflammatory and anti-infective purposes (e.g., in cystic fibrosis) (12, 13).

In parallel, the polymyxin class of antibiotics also serves as a critically important last-line agent. Polymyxins (PMB) are prototypical agents targeting the gram-negative membrane, and they function by using a detergent-like mechanism in targeting lipid A, an important anchor for lipopolysaccharide (LPS) on the outer membrane (14, 15). However, resistance to last-line agents, including polymyxin and ATM-based therapies, has forced clinicians to consider alternative and understudied strategies.

With growing resistance to last-line agents such as ATM and PMB (16–18), bacteriophages (phages) have been proposed as a solution. Discovered over a century ago, phages are viruses that infect and kill bacteria and have long been of interest as potential antibacterial agents (19, 20). Phages have gained renewed attention as an orthogonal therapy for combating antimicrobial resistance in *P. aeruginosa* (19, 20) due to their safety profile, specificity, and rapid bactericidal action. Phages can disrupt the extracellular matrix, penetrate biofilms, and directly lyse bacterial cells (21). *Pseudomonas* phages have been found to infect *P. aeruginosa* by recognizing specific cell surface receptors including LPS, type-IV pilus, and porins (20, 22, 23).

LPS is among the most common *Pseudomonas* phage receptors and is an overlapping target with existing antimicrobials (e.g., the polymyxins) (24). LPS plays a major role in the host immune response, tissue damage, antibiotic resistance, and biofilm formation. LPS comprises lipid A, core oligosaccharide, and the O-antigen. Together, these components form the outer leaflet of the outer membrane, which acts as a protective barrier limiting antibiotic permeability (25–27). LPS alterations associated with polymyxin resistance have been shown to increase membrane permeability (28, 29), potentially enhancing antibiotic influx and providing a rationale for combining phages with antibiotics (30).

*Pseudomonas* phages E215 (LPS-specific), PYO2 (LPS-specific), and LUZ19 (pili-targeted) have been previously characterized and shown to be useful in treating recalcitrant infections (22, 31). These phages have been characterized against a panel of isolates sourced from patients with cystic fibrosis (19, 31). All three phages utilize PAO1 as their domesticated, reference host, and have latent periods <25 min with burst sizes of 100–200 plaque-forming units per colony-forming units (PFU/CFU) (22).

Based on these observations, we hypothesized that LPS-specific phage would drive the emergence of phage-resistant subpopulations with altered membrane permeability, resulting in collateral sensitivity to beta-lactams in a drug-dependent manner. To test this, we first established proof-of-concept phage-antibiotic synergy using a clinically relevant cocktail of E215 and LUZ19 against the domesticated reference host PAO1 under dynamic dosing conditions in the Hollow Fiber Infection Model (HFIM), building on prior phage characterization in this strain (32). Both phages have been utilized in compassionate use cases, and the inclusion of E215 and LUZ19 reflects a deliberate extension of prior phage characterization work (32, 33). PYO2 was not included in the HFIM as it shares the same O-antigen receptor target as E215, precluding independent phage quantification in combination. We then assessed if synergistic effects are extendable to a PYO2-sensitive clinical isolate and if synergy was beta-lactam-specific (33). In prior work, PYO2 was identified as the only phage among LUZ19, E215, and PYO2 with meaningful lytic activity against AR-0231 in both static time-kill and Hollow Fiber Infection Model studies, while LUZ19 and E215 monotherapy showed no bactericidal effect. Furthermore, combining PYO2 with LUZ19 against AR-0231 produced an antagonistic interaction, motivating the selection of PYO2 alone for the present study (33). We aimed to determine whether LPS-specific phage selects for a phage-resistant bacterial subpopulation with altered beta-lactam sensitivity. Using a previously published LC-MS/MS-based assay (10), we sought to characterize whether any changes in sensitivity were attributable to alterations in beta-lactam permeability in the phage-resistant subpopulation. This study is motivated by both the previous data on mechanisms of beta-lactam and polymyxin synergy and the ongoing efforts to optimally combine phage therapy with existing antimicrobials (34–37). In developing improved phage-antibiotic combination strategies, regimens can be more efficiently and strategically designed for *in vivo* or clinical studies.

## MATERIALS AND METHODS

### Bacteria, phages, antibiotics, and media

For the HFIM studies, laboratory *P. aeruginosa* PAO1 was utilized. For all time-kill and permeability studies, CDC AR-0231 was utilized. AR-0231 is a clinical, *Klebsiella pneumoniae* carbapenemase-5 (KPC-5)-producing XDR *P. aeruginosa* (*bla*_KPC5_, *bla*_OXA-2_, *bla*_OXA-50, and_ *bla*_PDC-103_; MIC_Aztreonam_ >64 µg/mL, MIC_Ceftazidime_ >64 µg/mL, MIC_Cefepime_ >32 µg/mL, MIC_Meropenem_ >8 µg/mL, MIC_Imipenem_ >64 µg/mL, and MIC_Piperacillin_ >128 µg/mL, FDA/CDC AR Bank) (38). Previously characterized (22) and clinically relevant lytic *Pseudomonas* phages E215 (LPS-specific), PYO2 (LPS-specific), and LUZ19 (pili-targeted) were freshly cultured, and filter-sterilized lysates were quantified prior to each experiment using PAO1 as the reference host.

E215 and LUZ19 were selected for the HFIM as both have been previously characterized against PAO1 and utilized in compassionate use cases (32). PYO2 was not assessed in combination with E215 in either experimental platform as both phages utilize the O-antigen as the receptor, precluding independent phage quantification and confounding the interpretation of combination effects. AR-0231 was selected for static time-kill and permeability studies as a clinically relevant XDR isolate, with PYO2 previously characterized as the only effective phage against this isolate (33). Beta-lactam permeability changes were not assessed in LUZ19-resistant mutants as wild-type AR-0231 was inherently resistant to LUZ19, precluding mutant generation. AR-0231 was previously studied against each of these phages in monotherapy and in dual-phage combination in the HFIM over 7 days (33). PYO2 monotherapy was the only regimen demonstrating sustained bactericidal activity, with phage resistance emerging under prolonged selective pressure, while the combination with LUZ19 resulted in antagonism, providing a robust rationale for the present study’s focus on PYO2 (33).

Polymyxin B sulfate (Sigma Aldrich), aztreonam (AKScientific), ceftazidime (Sigma Aldrich), cefepime (AKScientific), meropenem (AKScientific), imipenem (AKScientific), and piperacillin (AKScientific) were all aseptically prepared in saline on the day of each experiment. All experiments were conducted using cation-adjusted Mueller-Hinton broth (MHB) and samples enumerated by colony/plaque counting on Mueller-Hinton agar (MHA).

Bacteria were enumerated by serial dilution, plated using automated spiral plating (WASP Touch, Don Whitley), and enumerated by automatic colony counting (Protos, Synbiosis). Serial dilutions of samples prior to plating rendered any residual antibiotic or phage concentration negligible, and the consistency of colony counts across multiple dilutions confirmed the absence of drug or phage carryover effects. Phages were enumerated as previously described (32, 33), whereby 4 μL of serially diluted phages was spotted in quadruplicate on freshly prepared *P. aeruginosa* lawns using host isolate PAO1. Plaques were manually enumerated 8–16 h after plating.

### Hollow Fiber Infection Model

The 7-day HFIM experiments (Fig. S1) were performed using a system clearance rate of 0.042 L/h with a total volume of 125 mL (2 h half-life) and using the polyvinylidene fluoride fibers (0.1 μm, FiberCell, C2025), as previously described (32, 39).

ATM was dosed to simulate a 500-mg 2-h prolonged infusion every 8 h, corresponding to 25% of the standard 6 g/day total daily dose evaluated previously (11). This low-dose strategy was used because HFIM experiments utilized laboratory isolate PAO1, which is aztreonam-susceptible. Using a smaller clinical dose allowed assessment of the influence of PBP3-mediated filamentation in the ATM-sensitive PAO1, which has been reported previously as a potential mechanism for phage-beta-lactam synergy (40) and SOS induction (10, 41, 42).

Phages were administered via continuous infusion to a steady state of 10^2^ PFU/mL, the limit of quantification. This experimental design maximizes the dynamic range of detecting phage proliferation and to ensure that any detectable phage measured >10^2^ PFU/mL over 168 h could be attributable to endogenous phage production.

HFIM data generated from E215 continuous infusion, LUZ19 continuous infusion (CI), and E215 +LUZ19 continuous infusion were previously reported elsewhere but are recreated here for comparison with antibiotic-containing treatments (32). ATM-monotherapy as a 2-h prolonged infusion every 8 h (2hPI Q8H) and combination ATM 2hPI Q8H + LUZ19/E215 CI are newly generated data used to obtain a baseline assessment of phage-antibiotic synergy in the reference host, including morphological changes via scanning electron microscopy (SEM). Phage and antibiotic resistance evaluations and mutation analysis were not performed. HFIM experiments were conducted as single replicates.

### Scanning electron microscopy

Treatment-induced changes in bacterial morphology were examined following 2, 12, 26, and 168 h of exposure in the HFIM, corresponding to the expected period of maximal phage-mediated killing, bacterial rebound, regrowth, and the terminal HFIM time point. Samples were extracted from the HFIM and fixed in 2.4% glutaraldehyde (Electron Microscopy Sciences, Ref # 16020, USA), filtered onto 0.2-µm nucleopore polycarbonate membranes (Sigma-Aldrich, Ref # WHA10417006, Whatman, USA), and slowly dehydrated through a series of graded ethanol washes to preserve cell structures. Following the final wash, the dehydrated samples were treated with hexamethyldisilazane and allowed to air-dry. Prepared samples were gold sputter-coated and imaged using a Carl Zeiss Auriga focused ion beam scanning electron microscopy (SEM) at 10,000× magnification with a 2.0 kV accelerating voltage. Assembled image panels provided insights into the morphological alterations conferred by ATM and a 2-phage cocktail against PAO1.

### Static time-kill studies

To assess major anti-*Pseudomonal* antibiotics, *in vitro* static time-kill studies (STKS) were used to quantify the rate and extent of bacterial killing, with concordant phage proliferation. STKS were performed using Clinical and Laboratory Standards Institute standards in 20 mL volumes (43). Fresh overnight subcultures were resuspended in MHB and brought into the early-log phase over 1 h at 37°C. Extracellular beta-lactamases and debris were washed from the bacterial stock by centrifuging at 6,000 rcf for 6 min. Spectrophotometry (OD_620_) was used to set the starting inoculum to 10^7^ CFU/mL.

Aztreonam/avibactam and polymyxin B were examined as clinical monotherapy regimens and evaluated in combination with phage PYO2 against AR-0231. Antibiotics were dissolved in saline and filter-sterilized prior to each experiment. ATM/AVI was studied at 17/2.5 mg/L, 33/4.9 mg/L, 67/9.9 mg/L, and 132/19.8 mg/L (11). PMB was studied at 0.75, 1.5, 2.5, and 5 mg/L, based on Monte Carlo simulation of C_ss,avg_ (1.5 mg/L) from guideline dosing (44, 45). Phage stocks were quantified using PAO1 as the reference host. As AR-0231 was standardized to a starting inoculum of 10^7^ CFU/mL, phage concentrations based on PAO1 titers were used to achieve multiplicity of infection (MOI_input_) of +1, 0, and −1 (corresponding to 10^8^, 10^7^, and 10^6^ PFU/mL respectively). At each time point, both bacteria and phage were enumerated by plating on MHA. The full treatment matrix was evaluated in biological duplicates.

### Sequential time kill studies

Sequential combination STKS were designed based on findings from the simultaneous combination in standard STKS. Both treatment orders—PYO2-then-antibiotics and antibiotics-then-PYO2—were evaluated, with the first agent added at 0 h and the second at 8 h. The administration time of the second agent was based on the observed nadir (i.e., lowest observed bacterial concentration) in monotherapy. Compared to the standard STKS, a reduced treatment matrix was evaluated in biological duplicates. Beta-lactams were evaluated at their clinical free, maximum concentrations (*f*C_max_): 72.6 (PIP), 66.6/9.9 (ATM/AVI), 48.0 (MEM), and 66.4 mg/L (IPM). PMB was evaluated at the free, steady-state concentration (*f*C_ss,avg_), 1.55 mg/L, following guideline dosing principles (44, 46). All PYO2 starting concentrations were 10^7^ PFU/mL.

### Minimum inhibitory concentration

Minimum inhibitory concentrations (MICs) were determined for all six anti-*Pseudomonal* beta-lactams against AR-0231 and the PYO2-resistant mutant using broth microdilution as per CLSI guidelines. Two-fold serial dilutions of each antibiotic were prepared in cation-adjusted Mueller-Hinton broth and inoculated to a final concentration of 10^5.5^ CFU/mL. Plates were incubated at 37°C for 16–20 h, and the MIC was defined as the lowest concentration completely inhibiting visible bacterial growth.

### Phage-resistant mutant generation by serial passage

Phage-resistant mutants of AR-0231 were generated through static time-kill experiments (Fig. S2). PYO2 phage stock was titered before the time-kill experiment to ensure accurate phage counts. The host bacteria were grown overnight on Mueller-Hinton agar plates at 37°C. The following day, bacteria were grown to the log phase and then centrifuged with MHB for a total of three washes. The PYO2 stock was prepared at a concentration of 10^7^ PFU/mL. After the 24-h time point, the bacteria were streaked out on MHA plates and incubated overnight at 37°C. The following day, after the incubation period, a single colony was collected and cultured again. To evaluate the changes in anti-*Pseudomonal* beta-lactam activity, PYO2-resistant AR-0231 (AR0231-PYO2R) was studied in STKS and through quantification of beta-lactam permeability.

### Permeability assay

Outer membrane permeability was quantified for six anti-*Pseudomonal* beta-lactam antibiotics (ATM, CAZ, FEP, MEM, IPM, and PIP) using a previously developed LC-MS/MS-based assay (10, 47). Briefly, this assay quantifies drug degradation kinetics under two conditions: permeability-limited intact bacterial cells and enzyme kinetics-limited lysed bacterial cells to estimate drug-specific outer membrane permeability surface area coefficients. Degradation of each antibiotic was evaluated at starting concentrations of 30 mg/L, 10 mg/L, 3 mg/L, 1 mg/L, and 0.3 mg/L. LC-MS/MS standard curves utilized concentrations of 50 mg/L, 30 mg/L, 10 mg/L, 3 mg/L, 1 mg/L, and 0.3 mg/L. Quality control samples were evaluated at 40 mg/L, 4 mg/L, and 0.4 mg/L.

Bacteria were grown overnight in Mueller-Hinton broth at 37°C. Late-log phase bacterial suspensions were washed using five washing cycles in 25 mL PBS. After the final wash, the supernatant was collected to confirm minimal extracellular β-lactamase activity. The lysed matrix was generated by sonication of the bacterial suspension on ice at 14% power for 4 min using on/off cycles of 5 s duration. Samples from both intact and lysed matrices were plated and incubated overnight to confirm the final number of bacteria in suspension and to confirm the efficiency of sample sonication.

Samples were processed for analysis by LC-MS/MS by adding 100 µL of the sample to 400 µL of HPLC methanol containing an isotopically labeled internal standard. Deuterated internal standards were used at a concentration of 5 mg/L. For internal standards, ATM-d6 was used for aztreonam; MEM-d6 for MEM; FEP-d8 for FEP; CAZ-d5 for CAZ and IMI; and PIP-d5 for PIP (all sourced from LGC standards). Samples were then vortexed and centrifuged at 15,000 rpm at 4°C for 5 min. After centrifugation, 100 µL of the supernatant was combined with 900 µL of HPLC water for final analysis using multiple reaction monitoring, as previously described (10).

### Permeability coefficient determination by a mathematical model

Beta-lactam specific surface area permeability coefficients were estimated using a mathematical model described previously (10, 47). In intact cells, the model describes beta-lactam permeation from the extracellular space into the intracellular space after initial inoculation and then hydrolysis by beta-lactamase in the periplasmic space. In lysed cells, beta-lactams are exposed directly to beta-lactamase without requiring membrane penetration, owing to outer membrane disruption. In the lysed matrix, beta-lactam elimination is due to thermal degradation and enzymatic hydrolysis, only. For the drugs within the supernatant controls, thermal degradation is expected to be the only elimination route.

For aztreonam, ceftazidime, cefepime, imipenem, and piperacillin:

Intact cells:


$$V_{extra}\cdot \frac{dC_{extra}}{dt}=CL_{D}\cdot (C_{intra}-C_{extra})-CL_{deg}\cdot C_{extra}$$

$$V_{intra}∙\frac{dC_{intra}}{dt}={CL}_{D}∙\left(C_{extra}-C_{intra}\right)-CL∙C_{intra}$$

Lysed cells:


$$V_{dist}∙\frac{dC_{lysed}}{dt}=-{CL}_{deg}∙C_{lysed}-0.05∙CL∙C_{lysed}$$

Supernatant:


$$V_{dist}∙\frac{dC_{ctr}}{dt}=-{CL}_{deg}∙C_{ctr}$$

For meropenem:

Intact cells:


$$V_{extra}\cdot \frac{dC_{extra}}{dt}=CL_{D}\cdot (C_{intra}-C_{extra})-CL_{deg}\cdot C_{extra}$$

$$V_{intra}∙\frac{dC_{intra}}{dt}={CL}_{D}∙\left(C_{extra}-C_{intra}\right)-\frac{{CL}_{int}∙K_{m}}{K_{m}+C_{intra}}∙C_{intra}$$

Lysed cells:


$$V_{dist}∙\frac{dC_{lysed}}{dt}=-{CL}_{deg}∙C_{lysed}-\frac{\left({CL}_{int}∙K_{m}\right)∙0.05}{K_{m}+C_{lysed}}∙C_{lysed}$$

Supernatant:


$$V_{dist}∙\frac{dC_{ctr}}{dt}=-{CL}_{deg}∙C_{ctr}$$

The value 0.05 corrects for the decrease in beta-lactamase concentration after bacterial cells are lysed (10, 47). As previously described, the extracellular volume was fixed to 0.95 mL and the periplasmic space was fixed to 0.05 mL based on the previous model, which accounts for a total cellular space of 1 mL by assumption (10, 47).

The outer membrane permeability surface area coefficients (PC, nm/s) for beta-lactams, the key metric for drug-specific permeability, were calculated as a function of the model-estimated distribution clearance ${CL}_{D}$ (mL/min) (10, 47). We assumed a bacterial surface area of 3.7 m^2^ from Lakaye et al. (48), consistent with the previously established model (10, 47). Therefore, the average bacterial count of 2.14 × 10^9^ CFU/mL is equivalent to a surface area of 79.18 cm^2^. For PYO2 phage-resistant AR-0231, the average bacterial count of 9.95 × 10^8^ cells after five washes is equivalent to a surface area of 36.82 cm^2^. PYO2 resistance was modeled as a dichotomous covariate affecting the permeability to account for phage pretreatment effects in combinatorial therapy.


$$PC=\frac{{CL}_{d}∙10^{7}}{Surfacearea∙60}$$

Both linear elimination and Michaelis-Menten kinetics are tested for enzymatic degradation in the model as the primary goal was to capture drug degradation under enzyme kinetics-limited conditions. Handling of thermal degradation and unexpected hydrogen swapping is described in the Supplemental Materials. The elimination model for each beta-lactam was chosen based on the goodness-of-fit plots (Fig. S6 and S7), Akaike information criterion (AIC) values, and parameter estimation plausibility.

Simulations of the permeability model were performed based on the simulated unbound average steady-state beta-lactamase plasma concentrations (Fig. S8). The previously simulated unbound average steady-state plasma concentration for beta-lactamases in critically ill patients was used for the simulation of the intracellular concentration, except for piperacillin (47) (Table S1). The piperacillin unbound average steady-state plasma concentration is simulated from a previously published population pharmacokinetic model for piperacillin and tazobactam (49). The 10^7^ CFU/mL is used as the normalized bacterial population count for simulation to reflect the inoculum effect for the clinical *P. aeruginosa* (50). In all the simulated intracellular drug concentration profiles of beta-lactams, the PYO2-resistant AR-0231 strain had a higher level compared to the AR-0231 strain.

Data management and visualization for permeability modeling were conducted in R version 4.5.1 (R Core Team, http://www.r-project.org) and R Studio Workbench (R Studio Team, Boston, MA), respectively. The permeability model was developed in NONMEM (Version 7.5.0, ICON Development Systems, Gaithersburg, MD, USA) and Perl-Speaks NONMEM (PsN), (Version 5.3.1). All the permeability data for the control, intact, and lysed cells were fitted with the model using the first-order conditional estimation method with interaction (FOCEI) with the ADVAN13 subroutine. The Monte Carlo simulation based on the permeability model was performed by the Mrgsolve package in R (51).

The model-building process was guided by the objective function value (OFV) and diagnostic plots. The OFV-based metric AIC is used for the non-hierarchical models.

### Whole-genome sequencing and analysis

Whole-genome sequencing of AR0231-PYO2R was performed using the Illumina NovaSeq 6000 platform. Genomic DNA library was prepared according to standard Illumina protocols and sequenced to generate 150-bp paired-end reads. Trimmed FASTQ files were mapped to the wild-type AR-0231 draft genome contigs using BWA v0.7.19 with default parameters (52). Single-nucleotide variants (SNVs) were identified using FreeBayes v1.3.10 (https://github.com/freebayes/freebayes). To identify additional insertion sequence-mediated recombination events, assembled mutant contigs were aligned to the wild-type assemblies using Geneious Prime 2025 (53), followed by manual inspection. The raw reads of all genomes were deposited in NCBI BioProject PRJNA1308160.

### Statistical analysis

Linear regression was used to evaluate the effect of the treatment sequence on 24-h bacterial concentrations in the sequential time-kill studies, with the treatment order as the primary predictor. Synergy between phage and antibiotics in simultaneous time-kill studies was assessed using Bliss independence analysis, where the expected combination effect was calculated as E(A+B)=E(A)+E(B)-E(A)∙E(B), with E(A) and E(B) representing the fractional bacterial killing by each agent alone. Excess-over-Bliss (EOB) was calculated as the difference between the observed and expected combination effects, with a statistically significant positive EOB indicative of synergy (54). Bliss independence was selected as the primary metric for synergy assessment as PYO2 monotherapy approached the limit of detection at the nadir, precluding reliable CFU-based comparisons between combination and monotherapy regimens. All statistical analyses were performed in R version 4.5.1 (R Core Team, http://www.r-project.org) and R Studio Workbench (R Studio Team, Boston, MA).

## RESULTS

### Evaluating phage-antibiotic synergy in the HFIM

**Fig 1:**
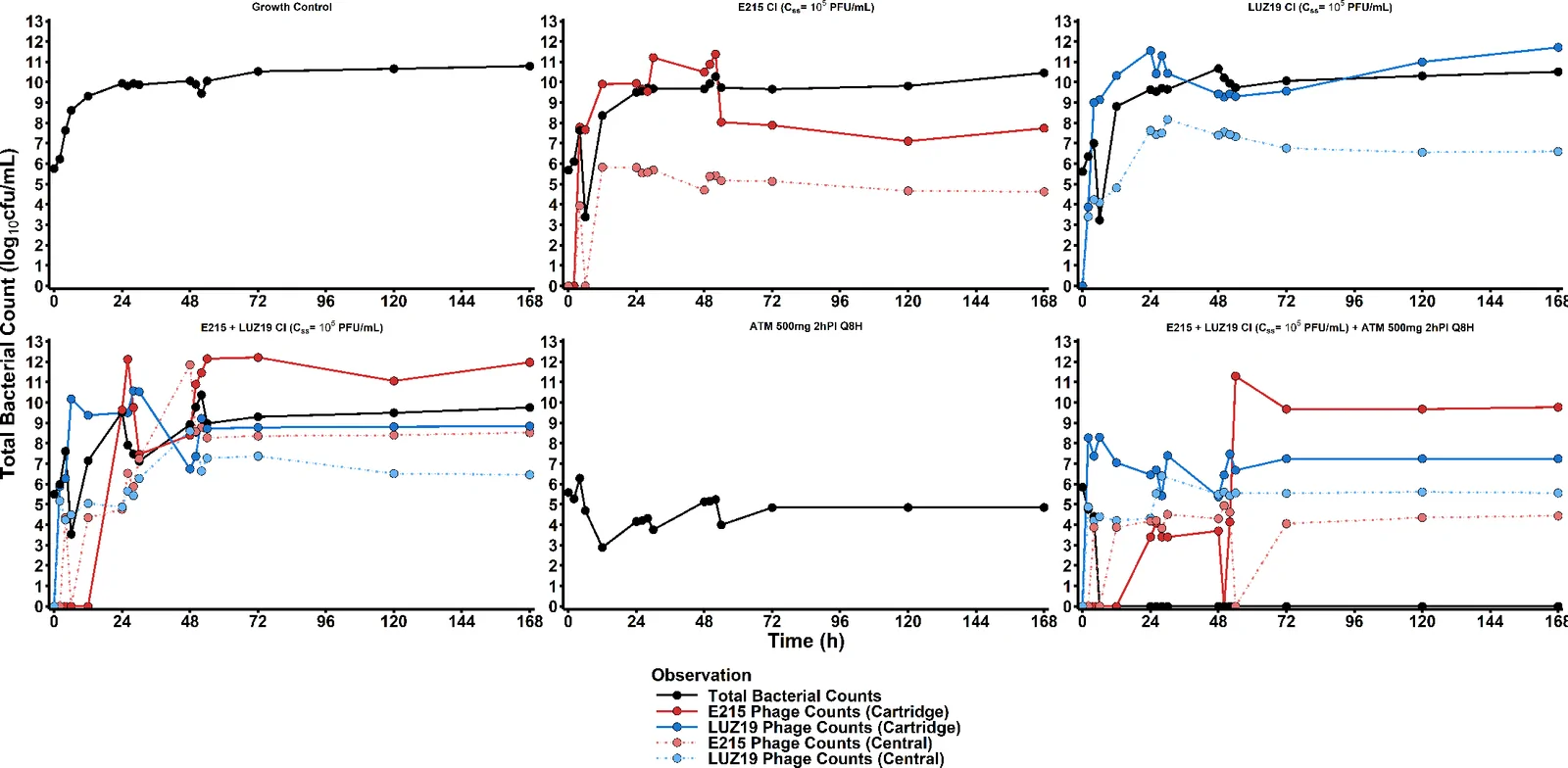
HFIM of LPS-specific phage, pili-targeted phage, aztreonam, and the combination. Comparison of phage monotherapy given as continuous infusion to an average steady-state concentration (Css,avg) of 102 PFU/mL, phage-phage combination therapy, aztreonam monotherapy as 2-h prolonged infusion every 8 h, and the combination of phage and antibiotics. Solid lines indicate bacteria and phage concentrations colocated in the HFIM cartridge, whereas dashed lines represent phage concentration in the HFIM central dosing compartment.

Monotherapy with either pili-targeted LUZ19 or LPS-specific E215 against laboratory host PAO1 resulted in a short period of rapid activity (Fig. 1), defined here as a bacterial reduction of >3 log_10_ CFU/mL, at 8 h followed by regrowth to the HFIM-carrying capacity by 24 h . When LUZ19 and E215 were combined, two distinct periods of bacterial killing occurred, first mediated by LUZ19 and then followed by E215. This finding is supported by the coinciding increases in HFIM cartridge (colocated with bacteria) LUZ19 concentration with delayed increases in E215 concentrations (55). Central compartment phage concentrations reached steady state as designed, reflecting the balance of continuous infusion and system clearance, while the substantially higher concentrations observed in the cartridge reflect local phage proliferation at the site of bacterial killing. Finally, low-dose aztreonam monotherapy (scaled to account for PAO1 being ATM-susceptible) resulted in bactericidal activity (3.40 log_10_ CFU/mL reduction), followed by sustained low-level counts throughout 168 h. When combined, LUZ19 +E215 +ATM resulted in rapid bactericidal activity followed by eradication of PAO1 by 6 h.

**Fig 2:**
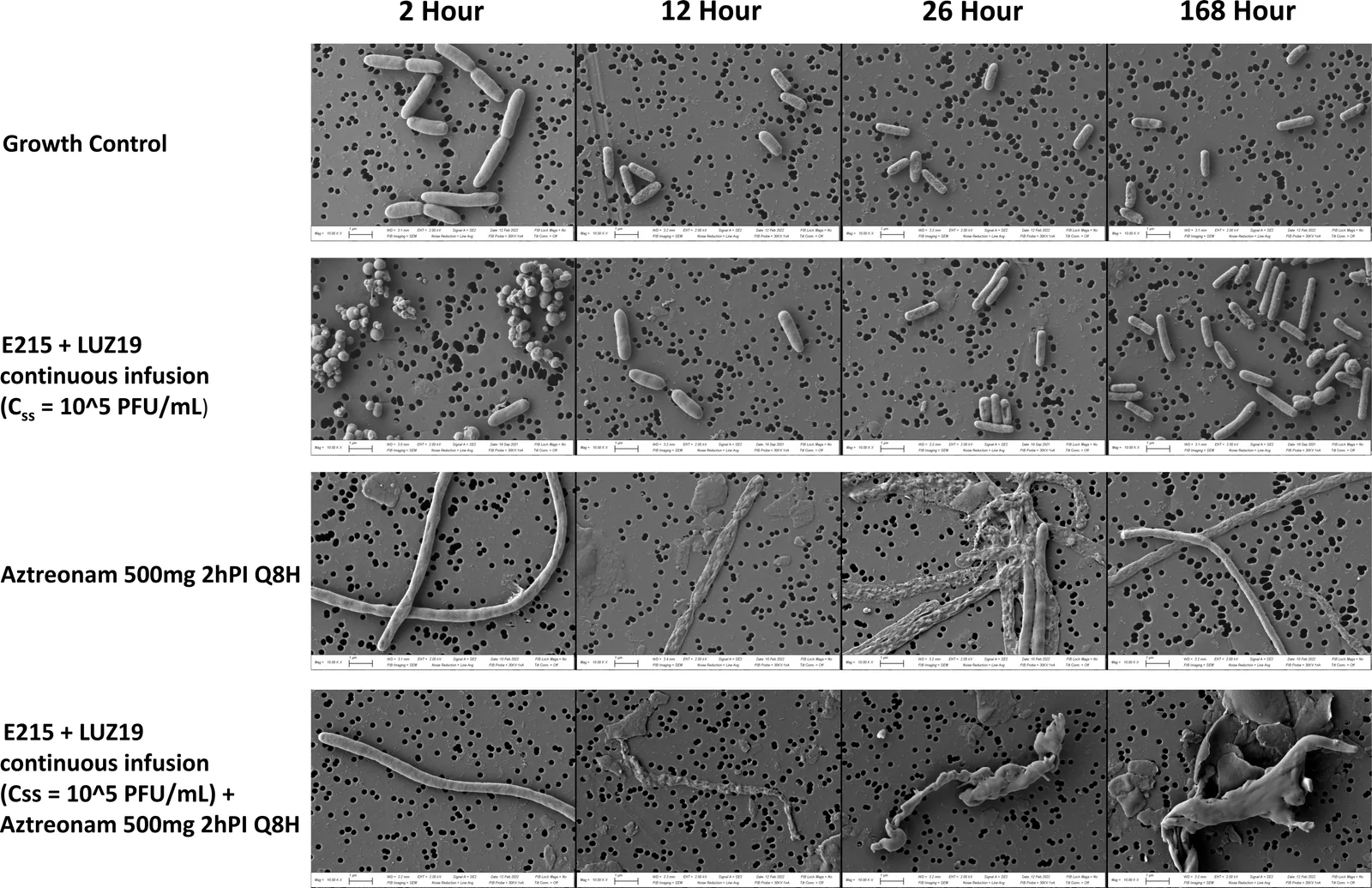
Scanning electron microscopy. SEM images of 2-phage cocktail, ATM, and ATM+2-phage cocktail at select time points. Data show pronounced filamentation with ATM-based therapy. Only debris was observable in the combination of phage with ATM.

Phenotypic changes previously correlated with bacterial rebound in the HFIM were assessed by time-series scanning electron microscopy. Images of the growth control and E215 +LUZ19 treatments revealed typical rod morphology, with an overall decrease in rod numbers following exposure to combination phage therapy (Fig. 2). ATM monotherapy induced a filamentous phenotype in PAO1, consistent with previous studies (56). SEM of the combination of ATM with phage showed only a sparse presence of filaments at 2 h, followed by debris from 12 to 168 h.

### Phage-antibiotic therapy versus KPC5-producing *P. aeruginosa*

**Fig 3:**
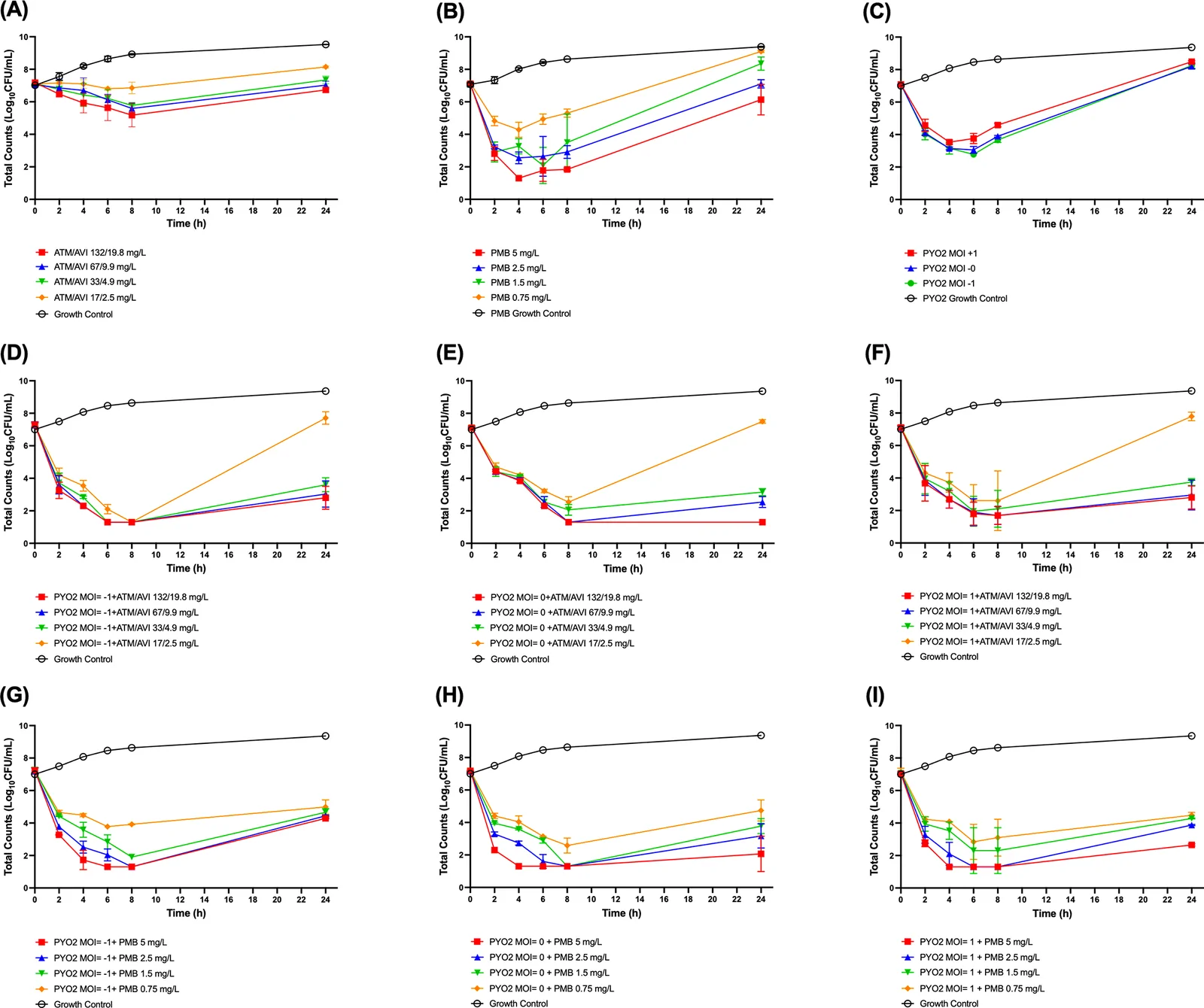
Simultaneous static time-kill experiments of LPS-specific phage versus last-line agents in KPC5-producing *P. aeruginosa*. (**A**) aztreonam/avibactam monotherapy. (**B**) Polymyxin B monotherapy. (**C**) PYO2 monotherapy. (**D**) Aztreonam/avibactam and PYO2 (MOIInput = −1) combination therapy. (**E**) Aztreonam/avibactam and PYO2 (MOIInput = 0) combination therapy. (**F**) Aztreonam/avibactam and PYO2 (MOIInput = +1) combination therapy. (**G**) Polymyxin B and PYO2 (MOIInput = −1) combination therapy. (**H**) Polymyxin B and PYO2 (MOIInput = 0) combination therapy. (**I**) Polymyxin B and PYO2 (MOIInput = +1) combination therapy. MOIinput represents the log-transformed input multiplicity of infection, corresponding to 10−1, 100, and 101 PFU/CFU for MOIinput of -1, 0, and +1, respectively.

Having established phage-antibiotic synergy in the susceptible laboratory strain, we next evaluated whether these findings extend to a clinically relevant XDR isolate AR-0231 using simultaneous static time-kill studies (33). Triplicate STKS with ATM/AVI, PMB, and LPS-specific PYO2 phage against the AR-0231 isolate showed expectedly pronounced bacterial killing effects for all three agents in monotherapy (Fig. 3). PMB showed the strongest antibacterial activity, with the highest concentration (5 mg/L), reducing bacterial counts by 5.82 log_10_ CFU/mL from baseline at the nadir. By comparison, ATM/AVI produced a reduction of 2.07 log_10_ CFU/mL from baseline at the nadir at its highest concentration. PYO2 monotherapy reduced bacterial counts by 3.97 log_10_ CFU/mL from baseline at the nadir, an intermediate effect relative to PMB and ATM/AVI, with little effect of MOI_Input_ on bacterial killing.

When antibiotics were combined with the *P. aeruginosa* phage PYO2, combination therapy outperformed antibiotic monotherapy in achieving maximal bacterial reduction (Fig. 3). When aztreonam/avibactam was combined with PYO2, bacterial reductions reached ≥5.98 log_10_ CFU/mL at concentrations ≥67/9.9 within the first 8 h at the lowest MOI_Input_. Bliss independence models of synergy use fewer assumptions about the underlying biology, and analysis showed that both ATM/AVI and PMB exhibited a statistically significant excess-over-bliss indicative of synergy (Fig. S3).

### Effects of bidirectional subpopulation selection on phage-antibiotic synergy

**Fig 4:**
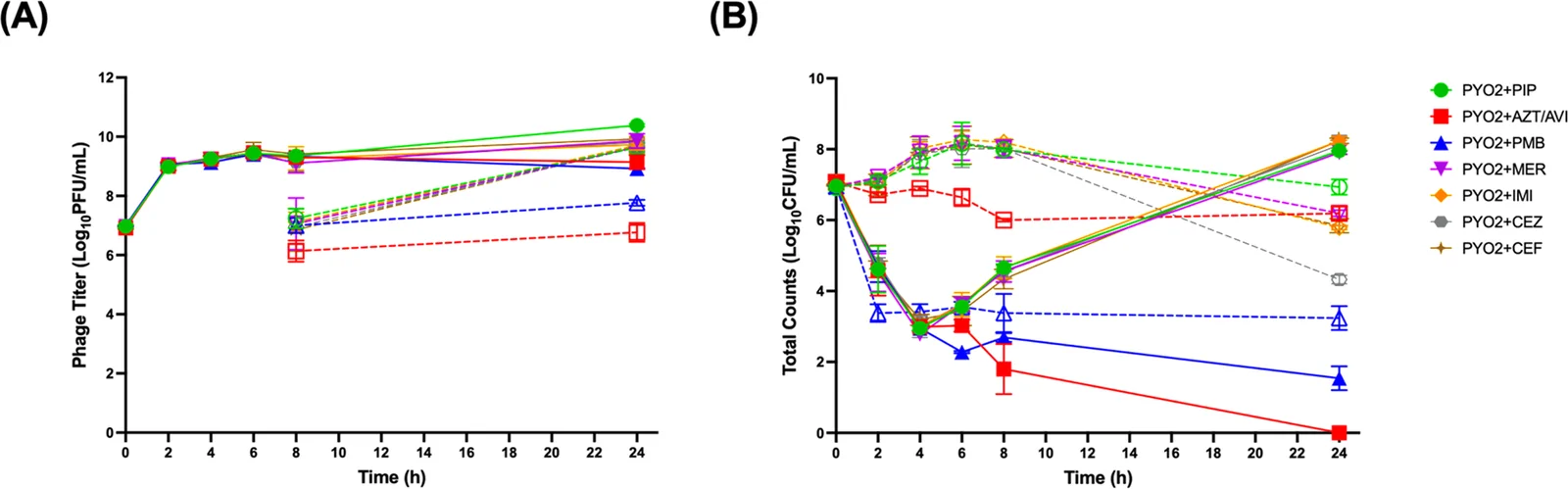
Evaluation of phage-antibiotic interactions by drug and dose sequence. (**A**) Phage amplification during the time-kill experiment. (**B**) Bacterial counts for the time-kill experiment. In both panels **A** and **B**, means are represented by observed points and standard deviations are represented by error bars for the experiment duplicate. The solid lines represent the combination treatment group with antibiotics added at 8 h, while the dashed lines represent the combination treatment group with phage added at 8 h.

We hypothesized that because of the unique mechanisms of action between LPS-specific phage and beta-lactams, synergy was resulting from subpopulation selection. To evaluate this, sequential STKS against wild-type AR-0231 were performed using a staggered dosing approach. We staggered the administration of either PYO2 or antibiotics to target the nadir of each regimen, which would represent a time point in the profile where the bacterial population is mostly composed of the resistant subpopulation. (Fig. 4) Since the nadir of AR-0231 bacterial counts was observed at 8 h, this was used as the time point for administration of the subsequently administered agent. For all antibiotics, the 24-h bacterial concentrations were reduced by phage-first dosing in a statistically significant manner (linear regression, β_phage-first_ = −6.19 log_10_ CFU/mL, *P* < 0.05), whereas antibiotic-first dosing failed given the baseline resistance profile of this isolate. PMB, which also targets LPS, showed improved activity, though the improvement in killing was less pronounced than that observed with beta-lactams.

### Effects of PYO2 resistance on beta-lactam permeability

Given the preferential benefit of phage-first treatment on beta-lactam pharmacodynamics and that PYO2 resistance is due to LPS modifications, we hypothesized that beta-lactams must be experiencing increased permeability in the PYO2-resistant subpopulation of AR-0231.

**Fig 5:**
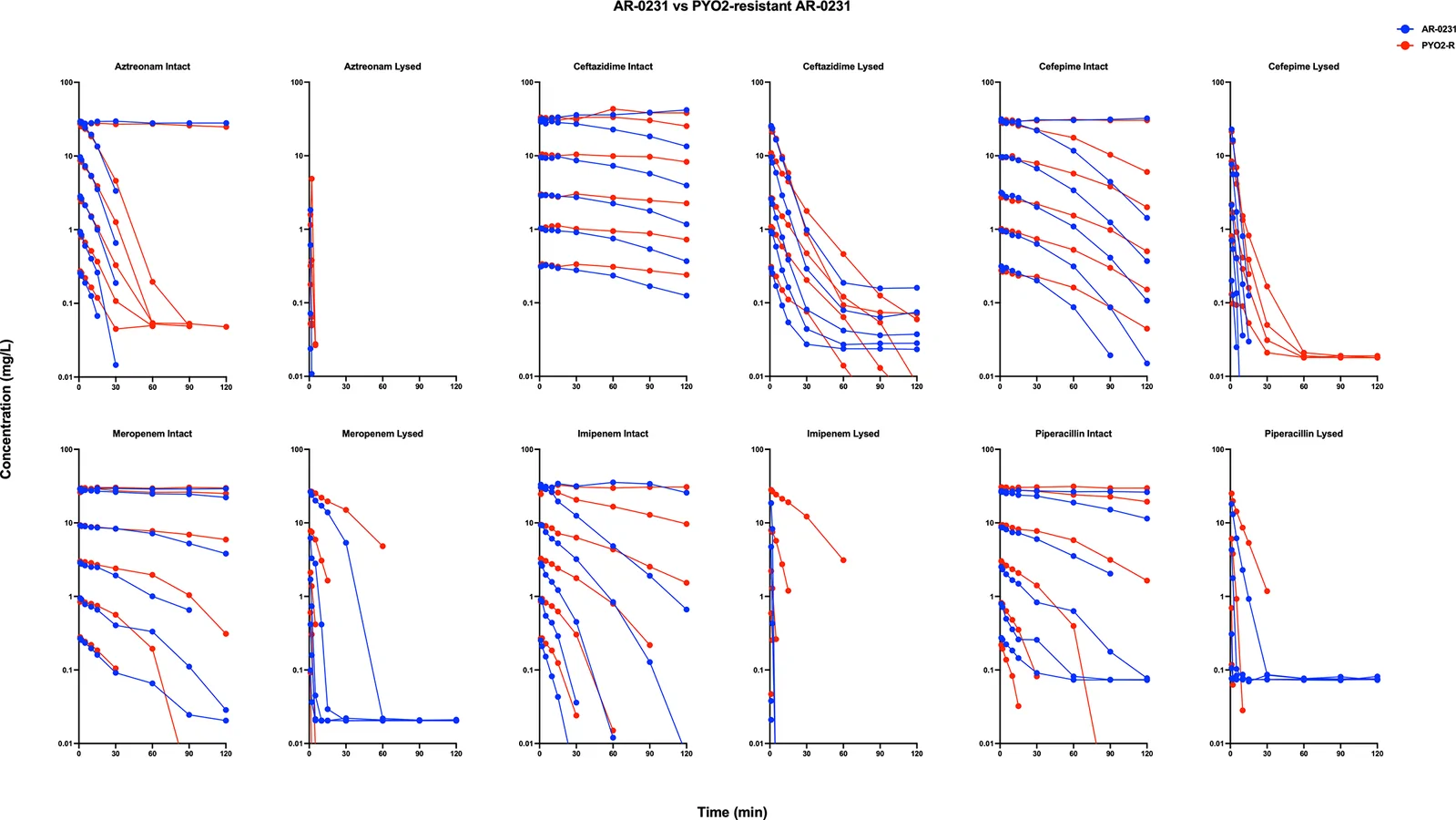
Quantifying beta-lactam permeability. Beta-lactam degradation kinetics were modeled between different matrices, containing permeability-limited intact bacterial cells, enzyme kinetic-limited lysed bacterial cells, and a negative supernatant control confirming extracellular enzyme removal. Comparison between the wild-type and resistant mutant permits calculation of permeability surface area coefficients for each beta-lactam.

To test this, we utilized LC-MS/MS analysis of drug concentrations over time for AR-0231 with the six beta-lactams (Fig. 5). Concentrations were measured under permeability-limited (intact membrane) and enzyme kinetics-limited (lysed membrane) conditions to estimate drug-specific permeability coefficients by mathematical modeling. (Fig. S4 to S7).

The results (Table 1) showed that aztreonam had the highest baseline permeability of 159 nm/s (4.6% residual standard error [%RSE]) compared to cephalosporins (ceftazidime and cefepime) and carbapenems (imipenem and meropenem). Notably, piperacillin demonstrated permeability comparable to that of cephalosporins and carbapenems, with an 87% increase (34.3%RSE) following PYO2 pretreatment, suggesting potential as a phage-beta-lactam combination candidate. Although aztreonam also demonstrated a significant increase in permeability when used in combination with phage, its rapid hydrolysis may be an important factor limiting its PBP target attainment in the periplasm of the AR-0231 strain unless combined with an effective beta-lactamase inhibitor (e.g., avibactam). For all beta-lactams, simulation studies predicted that the increased permeability could result in increased effects at clinically relevant free steady-state concentrations. (Table S1; Fig. S7)

**TABLE 1:** Permeability model estimation results^*a*^

Parameter estimations (RSE%)		Description	Drug					
			ATM	FEP	CAZ	IPM	MEM	PIP
Permeability	PC (nm/s)	Permeability coefficient of antibiotics across the outer membrane	159 (4.6%)	46.6 (3.8%)	20.2 (6.5%)	22.1 (7.3%)	6.32 (8.7%)	20.2 (6.3%)
	F (−)	Fold change of permeability for PYO2-resistant AR-0231 relative to wild-type	1.97 (10.4%)	1.26 (25.4%)	1.42 (31.9%)	2.83 (18.4%)	1.38 (27%)	1.87 (34.3%)
Enzymatic hydrolysis	CL (mL/min)	First-order hydrolyzed clearance of lactams	31.9 (3.6%)	2.85 (3.1%)	1.11 (2.4%)	0.893 (3.2%)	NA	2.76 (3.9%)
	CL_int_ (mL/min)	Michaelis-Menten intrinsic clearance of beta-lactamases	NA	NA	NA	NA	10.8 (15.9%)	NA
	Km (mg/L)	Michaelis-Menten rate constant	NA	NA	NA	NA	1.66 (17.3%)	NA
Residual error	σ_prop_ (CV%)	Proportional errors (%)	24.0 (7.5%)	31.8 (5.3%)	27.4 (5.0%)	42.8 (6.2%)	0 (fixed)	49.1 (5.7%)
	σ_add_ (mg/L)	Additive errors (mg/L)	0.0386 (16.6%)	0.0199 (43.2%)	0.0379 (17.3%)	0.136 (30.9%)	2.322 (4.8%)	0.0226 (13.9%)

^*a*^ ATM, aztreonam; FEP, cefepime; CAZ, ceftazidime; IPM, imipenem; MEM, meropenem; PIP, piperacillin.

### Increased sensitivity of PYO2-resistant KPC5-producing *P. aeruginosa* to beta-lactam monotherapy

**Fig 6:**
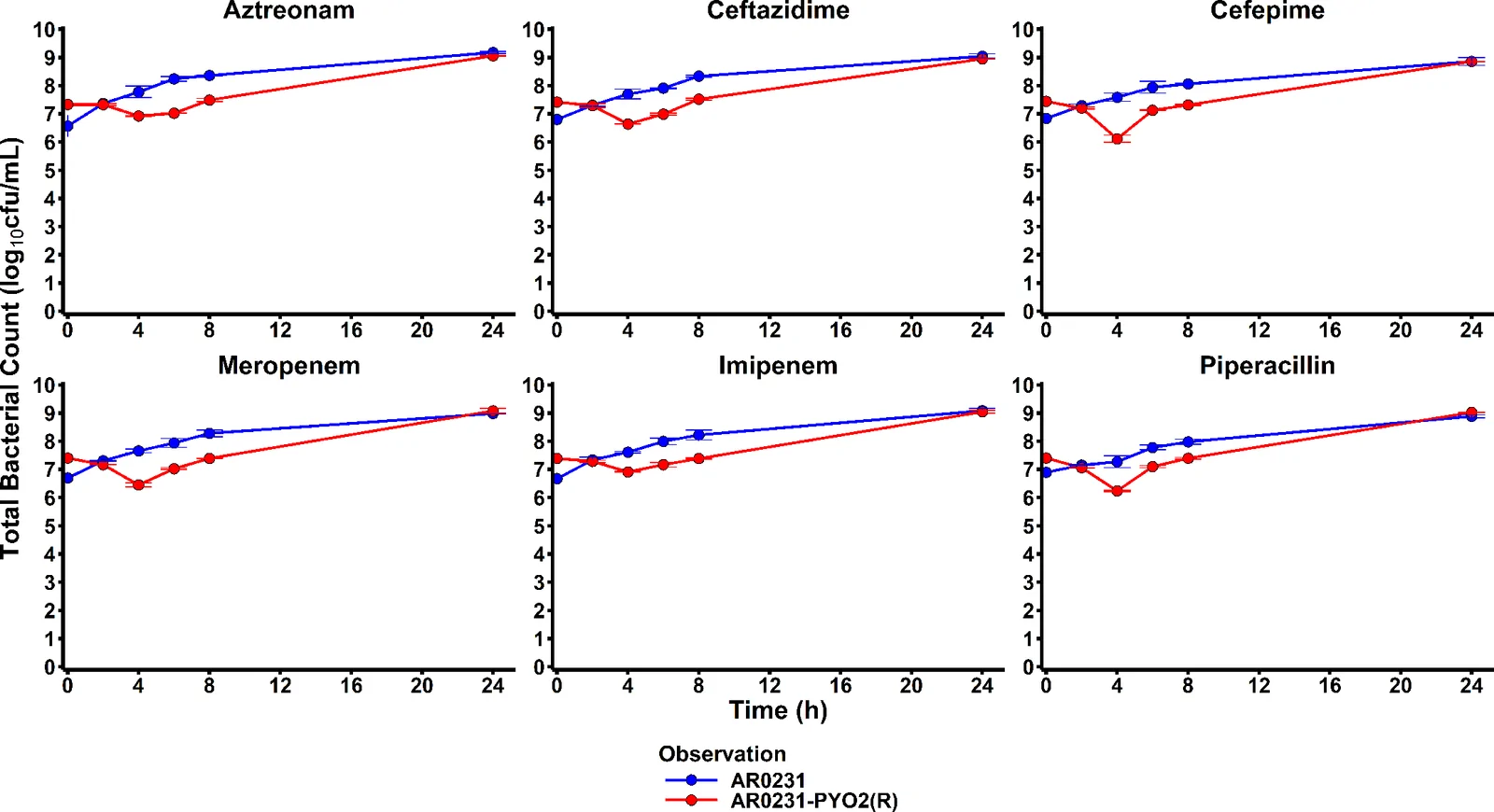
. Confirmatory enhancement of bacterial killing. As a secondary method to validate the LC-MS/MS findings of increased permeability, 30 mg/L of each beta-lactam (highest concentration used in permeability assay) was evaluated against both the wild-type AR-0231 and AR0231-PYO2R. These results suggest that enhanced permeability does improve the standalone activity of each anti-Pseudomonal beta-lactam tested. However, because of continued KPC-5 production, coformulation with beta-lactamase inhibitors would still be required to maximize antibacterial effects from the beta-lactams when combined with these LPS-specific phages.

To validate increased antibiotic sensitivity in PYO2-resistant, KPC5-producing *P. aeruginosa*, we performed STKS at 30 mg/L, the highest concentration tested in the permeability assay. (Fig. 6) At the nadir, AR0231-PYO2R demonstrated a 1.06 log_10_ CFU/mL average reduction compared to wild-type AR-0231 across all six beta-lactams tested. Pre- and post-experiment susceptibility are reported in Table S2. Because these agents remain KPC5 substrates, full susceptibility was not expected. However, these results were consistent with the permeability findings, supporting a trend toward increased beta-lactam susceptibility in the PYO2-resistant subpopulation.

### Whole-genome sequencing

To elucidate the genetic changes underlying the increased susceptibility to β-lactams in AR0231-PYO2R, we performed whole-genome sequencing. Comparative analysis identified a total of 10 SNPs/indels between AR0231-PYO2R and the wild type AR-0231, with no evidence of IS-mediated recombination. Among these variants, two mutations were mapped to genes involved in lipopolysaccharide biosynthesis. A frameshift mutation, caused by a single cytosine deletion, was identified in the O-antigen chain length regulator gene (*wzz*), which is expected to disrupt proper O-antigen modal length distribution (57). In addition, a nonsynonymous Leu294Arg substitution was detected in the α−1,6-rhamnosyltransferase migA, an enzyme involved in LPS core oligosaccharide modification (58, 59). Five additional mutations (four SNPs and one deletion) were identified in a gene encoding a hypothetical protein of unknown function, while the remaining three SNPs were located in intergenic regions. Given the lack of functional annotation and the absence of known roles in cell envelope structure or antibiotic resistance, these seven mutations are less likely to contribute significantly to the observed phage resistance or β-lactam susceptibility.

## DISCUSSION

The current proliferation of extensively drug-resistant ESKAPE pathogens, such as *P. aeruginosa*, demands new therapeutic strategies. Interest in phage-antibiotic combinations has been rekindled, with the hopes of leveraging synergistic mechanisms between phages and antibiotics to provide patients with a cure. In our experiments, known filamentous phenotypes emerged with ATM monotherapy, consistent with PBP3-mediated filamentation associated with bacterial rebound and phage-antibiotic synergy (56). Filamentation increases the biomass of individual bacterial cells, potentially enhancing available resources for phage replication and improving phage-bacteria encounter rates, which may contribute to the synergistic activity observed with aztreonam-containing regimens (40, 60, 61). Notably, persistently elevated phage concentrations were observed in the HFIM cartridge despite bacteria remaining below the limit of detection (10^2^ CFU/mL), consistent with a residual bacterial subpopulation sufficient to sustain phage replication through predator-prey dynamics given the exponential relationship between burst size and phage output (62). ATM in combination with phage was synergistic in these HFIM studies based on humanized concentration levels.

As clinical phage therapy strategies continue to be evaluated, the influence of subpopulation selection on collateral resistance and sensitivity must be better characterized. In this case, collateral sensitivity (63–65) is defined as the case where one anti-bacterial agent (i.e., phage) induces a selective pressure such that the remaining cells selected by the phage have an altered genotype or phenotype that results in sensitivity to an alternative anti-bacterial (i.e., beta-lactams). Combining multiple LPS-specific phages that exhibit different selective pressures on the O-antigen chain length in a strategic manner could make the synergy with beta-lactams more predictable.

Subsequent STKS findings suggested that the phage-resistant subpopulations exhibited a trend toward enhanced antibiotic sensitivity, consistent with previous sequential dosing experiments (9, 66). As PYO2 utilizes LPS as a receptor, we hypothesized that the synergy was due to enhanced outer membrane permeability in the PYO2-resistant subpopulation. These results support the consideration of initial, high-dose phage therapy just prior to traditional antimicrobials in the unique circumstances that might permit this to be done ethically (e.g., certain chronic infection cases).

Using a previously validated LC-MS/MS-based assay (10), all six anti-*Pseudomonal* beta-lactams had an estimated >25% increase in their permeability surface area coefficients for the PYO2-resistant mutants (Fig. 5; Table 1). The functional impact of increased permeability on bacterial killing was further supported by comparative time-kill studies between wild-type and PYO2-resistant bacteria, with results suggesting a trend toward enhanced susceptibility. Aztreonam had both high baseline permeability and a significant improvement in permeability in the resistant subpopulations, reinforcing ATM combination use with phage, especially with next-generation beta-lactamase inhibitors (e.g., AVI).

Mechanistically, PYO2 resistance in PAO1 likely arises through chromosomal mutations leading to truncated O-antigen (20, 22). Whole-genome sequencing of AR-0231 shows that PYO2 resistance is due to a frameshift deletion of *wzz*. Both *wzz* and migA regulate LPS structure, with *wzz* controlling the O-antigen chain length distribution and migA contributing to LPS core oligosaccharide modification. Alterations in these genes may, therefore, contribute to the increased outer membrane permeability and enhanced β-lactam susceptibility observed in AR0231-PYO2R (67). These findings indicate that O-antigen modifications resulting from PYO2 resistance may also improve target site access for lipid A-targeted polymyxins. However, the precise contributions of these mutations require further investigation. This mechanistic evidence suggests these findings may extend to PMB and colistin as alternatives to beta-lactam-based therapy.

Several limitations of this study warrant consideration. The absence of host immune responses, which would be expected to enhance bacterial (and phage) clearance *in vivo*, represents an inherent constraint of both experimental systems used (67). During HFIM experiments, aztreonam was administered at 25% of standard total daily dose to enable assessment of PBP3-mediated filamentation without antibiotic-driven eradication; however, it does not reflect standard clinical exposures. Clinical translation is better reflected by the XDR AR-0231 experiments conducted under static conditions for mechanistic exploration. Differences in pharmacokinetic complexity between the HFIM and STKS may limit direct comparison of findings across experiments but allowed for capture of clinically relevant exposure-response relationships and mechanistic exploration. Simulations of intracellular pharmacokinetics using human clinical drug concentrations suggest that the observed synergy may be clinically relevant (Fig. S8). Therefore, validation of these findings using *in vivo* infection models would improve translatability.

As phage-antibiotic combinations advance toward mainstream clinical use, strategic approaches such as dose sequencing offer a viable means to maximize the therapeutic benefit. Ultimately, the future of phage therapy, whether as monotherapy or in combination with antibiotics, depends on enhanced understanding of subpopulation dynamics, drug-drug interactions, and translational pharmacokinetic/pharmacodynamic approaches.

## SUPPLEMENTAL MATERIAL

Supplemental material aac.00048-26-s0001.pdf Supplemental methods; Fig. S1 to S8; Tables S1 and S2.

ASM does not own the copyrights to Supplemental Material that may be linked to, or accessed through, an article. The authors have granted ASM a non-exclusive, world-wide license to publish the Supplemental Material files. Please contact the corresponding author directly for reuse.

## References

[1] Salam MA, Al-Amin MY, Salam MT, Pawar JS, Akhter N, Rabaan AA, Alqumber MAA. 2023. Antimicrobial resistance: a growing serious threat for global public health. Health Care (Don Mills) 11:1946. doi:10.3390/healthcare11131946 PMC10340576 37444780

[2] Aslam B, Wang W, Arshad MI, Khurshid M, Muzammil S, Rasool MH, Nisar MA, Alvi RF, Aslam MA, Qamar MU, Salamat MKF, Baloch Z. 2018. Antibiotic resistance: a rundown of a global crisis. Infect Drug Resist 11:1645–1658. doi:10.2147/IDR.S173867 PMC6188119 30349322

[3] de la Fuente-Nunez C, Cesaro A, Hancock REW. 2023. Antibiotic failure: beyond antimicrobial resistance. Drug Resist Updat 71:101012. doi:10.1016/j.drup.2023.101012 PMC12224857 37924726

[4] Harms A, Maisonneuve E, Gerdes K. 2016. Mechanisms of bacterial persistence during stress and antibiotic exposure. Science 354:aaf4268. doi:10.1126/science.aaf4268 27980159

[5] Miller WR, Arias CA. 2024. ESKAPE pathogens: antimicrobial resistance, epidemiology, clinical impact and therapeutics. Nat Rev Microbiol 22:598–616. doi:10.1038/s41579-024-01054-w PMC13147291 38831030

[6] Diggle SP, Whiteley M. 2020. Microbe profile: *Pseudomonas aeruginosa*: opportunistic pathogen and lab rat. Microbiology 166:30–33. doi:10.1099/mic.0.000860 PMC7273324 31597590

[7] Reynolds D, Kollef M. 2021. The epidemiology and pathogenesis and treatment of *Pseudomonas aeruginosa* infections: an update. Drugs 81:2117–2131. doi:10.1007/s40265-021-01635-6 PMC8572145 34743315

[8] CDS. 2019. Antibiotic resistance threats in the United States 2019. Atlanta US Department of Health and Human Services CDC

[9] National Center for Emerging and Zoonotic Infectious Diseases (NCEZID), Centers for Disease Control and Prevention. 2024. Antimicrobial Resistance Threats in the United States, 2021–2022

[10] Smith NM, Boissonneault KR, Chen L, Petraitis V, Petraitiene R, Tao X, Zhou J, Lang Y, Kavaliauskas P, Bulman ZP, Holden PN, Cha R, Bulitta JB, Kreiswirth BN, Walsh TJ, Tsuji BT. 2022. Mechanistic insights to combating NDM- and CTX-M-Coproducing *Klebsiella pneumoniae* by targeting cell wall synthesis and outer membrane integrity. Antimicrob Agents Chemother 66:e0052722. doi:10.1128/aac.00527-22 PMC9487485 35924913

[11] Lodise TP, Smith NM, O’Donnell N, Eakin AE, Holden PN, Boissonneault KR, Zhou J, Tao X, Bulitta JB, Fowler VG Jr, Chambers HF, Bonomo RA, Tsuji BT. 2020. Determining the optimal dosing of a novel combination regimen of ceftazidime/avibactam with aztreonam against NDM-1-producing Enterobacteriaceae using a hollow-fibre infection model. J Antimicrob Chemother 75:2622–2632. doi:10.1093/jac/dkaa197 PMC8444334 32464664

[12] Elson EC, Mermis J, Polineni D, Oermann CM. 2019. Aztreonam lysine inhalation solution in cystic fibrosis. Clin Med Insights Circ Respir Pulm Med 13:1179548419842822. doi:10.1177/1179548419842822 PMC6463232 31019373

[13] Kirkby S, Novak K, McCoy K. 2011. Aztreonam (for inhalation solution) for the treatment of chronic lung infections in patients with cystic fibrosis: an evidence-based review. Core Evid 6:59–66. doi:10.2147/CE.S11181 PMC3195667 22022288

[14] Slingerland CJ, Kotsogianni I, Wesseling CMJ, Martin NI. 2022. Polymyxin stereochemistry and its role in antibacterial activity and outer membrane disruption. ACS Infect Dis 8:2396–2404. doi:10.1021/acsinfecdis.2c00307 PMC9745799 36342383

[15] Mohapatra SS, Dwibedy SK, Padhy I. 2021. Polymyxins, the last-resort antibiotics: mode of action, resistance emergence, and potential solutions. J Biosci 46:85. doi:10.1007/s12038-021-00209-8 PMC8387214 34475315

[16] Li Z, Cao Y, Yi L, Liu JH, Yang Q. 2019. Emergent polymyxin resistance: end of an era? Open Forum Infect Dis 6. doi:10.1093/ofid/ofz368 PMC6767968 31420655

[17] Yang S, Wang H, Zhao D, Zhang S, Hu C. 2024. Polymyxins: recent advances and challenges. Front Pharmacol 15:2024. doi:10.3389/fphar.2024.1424765 PMC11224486 38974043

[18] Barman J, Biswas P, Roy P, Hazra S. 2026. A divergent MBL-Fold Metallo-Hydrolase (Kmh-1) from *Klebsiella pneumoniae* confers aztreonam degradation and expands the β-Lactamase landscape. ACS Infect Dis. doi:10.1021/acsinfecdis.5c01093 42153452

[19] Forti F, Roach DR, Cafora M, Pasini ME, Horner DS, Fiscarelli EV, Rossitto M, Cariani L, Briani F, Debarbieux L, Ghisotti D. 2018. Design of a broad-range bacteriophage cocktail that reduces *Pseudomonas aeruginosa* biofilms and treats acute infections in two animal models. Antimicrob Agents Chemother 62:e02573-17. doi:10.1128/AAC.02573-17 PMC5971607 29555626

[20] Yu Z, Luong T, Banuelos S, Sue A, Ryu H, Segal R, Roach DR, Huang Q. 2024. Leveraging mathematical modeling framework to guide regimen strategy for phage therapy. PLoS Complex Syst 1:e0000015. doi:10.1371/journal.pcsy.0000015

[21] Racenis K, Lacis J, Rezevska D, Mukane L, Vilde A, Putnins I, Djebara S, Merabishvili M, Pirnay J-P, Kalnina M, Petersons A, Stradins P, Maurins S, Kroica J. 2023. Successful bacteriophage-antibiotic combination therapy against multidrug-resistant *Pseudomonas aeruginosa* left ventricular assist device driveline infection. Viruses 15:1210. doi:10.3390/v15051210 PMC10223274 37243293

[22] Forti F, Bertoli C, Cafora M, Gilardi S, Pistocchi A, Briani F. 2023. Identification and impact on *Pseudomonas aeruginosa* virulence of mutations conferring resistance to a phage cocktail for phage therapy. Microbiol Spectr 11:e0147723. doi:10.1128/spectrum.01477-23 PMC10714927 37966242

[23] Xuan G, Lin H, Kong J, Wang J. 2022. Phage resistance evolution induces the sensitivity of specific antibiotics in *Pseudomonas aeruginosa* PAO1. Microbiol Spectr 10:e0135622. doi:10.1128/spectrum.01356-22 PMC9603957 35972274

[24] Wang M, Kirienko NV. 2026. Lipopolysaccharide-targeting treatments for multidrug-resistant *Pseudomonas aeruginosa*. Expert Opin Ther Targets 30:203–211. doi:10.1080/14728222.2026.2639679 PMC13076032 41770946

[25] Verdial C, Serrano I, Tavares L, Gil S, Oliveira M. 2023. Mechanisms of antibiotic and biocide resistance that contribute to *Pseudomonas aeruginosa* persistence in the hospital environment. Biomedicines 11:1221. doi:10.3390/biomedicines11041221 PMC10135826 37189839

[26] Huszczynski SM, Lam JS, Khursigara CM. 2019. The role of *Pseudomonas aeruginosa* lipopolysaccharide in bacterial pathogenesis and physiology. Pathogens 9:6. doi:10.3390/pathogens9010006 PMC7168646 31861540

[27] Jurado-Martín I, Sainz-Mejías M, McClean S. 2021. *Pseudomonas aeruginosa*: an audacious pathogen with an adaptable arsenal of virulence factors. Int J Mol Sci 22:3128. doi:10.3390/ijms22063128 PMC8003266 33803907

[28] Delcour AH. 2009. Outer membrane permeability and antibiotic resistance. Biochim Biophys Acta 1794:808–816. doi:10.1016/j.bbapap.2008.11.005 PMC2696358 19100346

[29] Borrelli C, Douglas EJA, Riley SMA, Lemonidi AE, Larrouy-Maumus G, Lu W-J, Bonev BB, Edwards AM, Hoogenboom BW. 2025. Polymyxin b lethality requires energy-dependent outer membrane disruption. Nat Microbiol 10:2919–2933. doi:10.1038/s41564-025-02133-1 PMC12578643 41023239

[30] Akturk E, Oliveira H, Santos SB, Costa S, Kuyumcu S, Melo LDR, Azeredo J. 2019. Synergistic action of phage and antibiotics: parameters to enhance the killing efficacy against mono and dual-species biofilms. Antibiotics (Basel) 8:103. doi:10.3390/antibiotics8030103 PMC6783858 31349628

[31] Luong T, Kharrat L, Champagne-Jorgensen K, Melendez JA, Pride D, Conrad DJ, Roach DR. 2026. Ecological partitioning enables phage-antibiotic cooperation in a human *Pseudomonas infection*. Nat Commun 17:2615. doi:10.1038/s41467-026-69247-w PMC13003088 41667461

[32] Smith NM, Nguyen TD, Chin WH, Sanborn JT, de Souza H, Ho BM, Luong T, Roach DR. 2023. A mechanism-based pathway toward administering highly active N-phage cocktails. Front Microbiol 14:1292618. doi:10.3389/fmicb.2023.1292618 PMC10690594 38045026

[33] Nguyen TD, Sanborn JT, Ho BM, Luong T, Wood TD, Chen L, Roach DR, Smith NM. 2026. Pharmacodynamic individualization of phage therapy against a KPC-5-producing *Pseudomonas aeruginosa*. JAC Antimicrob Resist 8:dlaf257. doi:10.1093/jacamr/dlaf257 PMC12776353 41510198

[34] Aye SM, Galani I, Yu H, Wang J, Chen K, Wickremasinghe H, Karaiskos I, Bergen PJ, Zhao J, Velkov T, Giamarellou H, Lin YW, Tsuji BT, Li J. 2020. Polymyxin triple combinations against polymyxin-resistant, multidrug-resistant, KPC-Producing *Klebsiella pneumoniae*. Antimicrob Agents Chemother 64:e00246-20. doi:10.1128/AAC.00246-20 PMC7526826 32393492

[35] Mahadevan R, Garcia E, Sharma R, Qiu H, Elsheikh A, Parambi R, Abboud CS, Pasteran F, Ramirez MS, Kaye KS, Bonomo RA, Rao GG. 2026. A mechanism-based pharmacokinetic/pharmacodynamic analysis of polymyxin B-based combination therapy against carbapenem-resistant *Klebsiella pneumoniae* isolates with diverse phenotypic and genotypic resistance mechanisms. Antimicrob Agents Chemother 70:e0078225. doi:10.1128/aac.00782-25 PMC12888851 41416792

[36] Lenhard JR, Nation RL, Tsuji BT. 2016. Synergistic combinations of polymyxins. Int J Antimicrob Agents 48:607–613. doi:10.1016/j.ijantimicag.2016.09.014 PMC5237374 27865626

[37] Bergen PJ, Bulman ZP, Landersdorfer CB, Smith N, Lenhard JR, Bulitta JB, Nation RL, Li J, Tsuji BT. 2015. Optimizing polymyxin combinations against resistant gram-negative bacteria. Infect Dis Ther 4:391–415. doi:10.1007/s40121-015-0093-7 PMC4675771 26645096

[38] Lutgring JD, Machado MJ, Benahmed FH, Conville P, Shawar RM, Patel J, Brown AC. 2018. FDA-CDC antimicrobial resistance isolate bank: a publicly available resource to support research, development, and regulatory requirements. J Clin Microbiol 56:e01415-17. doi:10.1128/JCM.01415-17 PMC5786719 29118174

[39] Anonymous. 2013. AZACTAM (aztreonam for injection, USP) [package insert]. Bristol-Myers Squibb Company, Princeton, NJ.

[40] Bulssico J, PapukashvilI I, Espinosa L, Gandon S, Ansaldi M. 2023. Phage-antibiotic synergy: cell filamentation is a key driver of successful phage predation. PLoS Pathog 19:e1011602. doi:10.1371/journal.ppat.1011602 PMC10519598 37703280

[41] Blázquez J, Gómez-Gómez JM, Oliver A, Juan C, Kapur V, Martín S. 2006. PBP3 inhibition elicits adaptive responses in *Pseudomonas aeruginosa*. Mol Microbiol 62:84–99. doi:10.1111/j.1365-2958.2006.05366.x 16956383

[42] Smith NM, Boissonneault KR, Holden PN, Kaur JN, Klem JF, Cha R, Sutton MD, Tsuji BT. 2024. PBP-3 directed therapy in VIM-producing *Pseudomonas aeruginosa* creates bacterial transformers, persisters in disguise. Int J Antimicrob Agents 64:107260. doi:10.1016/j.ijantimicag.2024.107260 38945177

[43] Clinical and Laboratory Standards Institute. 1999. Methods for determining bactericidal activity of antimicrobial agents; approved guideline. Clinical and Laboratory Standards Institute, Wayne, PA.

[44] Tsuji BT, Pogue JM, Zavascki AP, Paul M, Daikos GL, Forrest A, Giacobbe DR, Viscoli C, Giamarellou H, Karaiskos I, Kaye D, Mouton JW, Tam VH, Thamlikitkul V, Wunderink RG, Li J, Nation RL, Kaye KS. 2019. International consensus guidelines for the optimal use of the polymyxins: endorsed by the American college of clinical pharmacy (ACCP), European society of clinical microbiology and infectious diseases (ESCMID), infectious diseases society of America (IDSA), international society for anti-infective pharmacology (ISAP), society of critical care medicine (SCCM), and society of infectious diseases pharmacists (SIDP). Pharmacotherapy 39:10–39. doi: 10.1002/phar.2209 10.1002/phar.2209 PMC7437259 30710469

[45] Sandri AM, Landersdorfer CB, Jacob J, Boniatti MM, Dalarosa MG, Falci DR, Behle TF, Bordinhão RC, Wang J, Forrest A, Nation RL, Li J, Zavascki AP. 2013. Population pharmacokinetics of intravenous polymyxin b in critically ill patients: implications for selection of dosage regimens. Clin Infect Dis 57:524–531. doi:10.1093/cid/cit334 23697744

[46] Anonymous. 2011. Polymyxin B for injection, USP [package insert]. Bedford Laboratories, Bedford, OH.

[47] Kim TH, Tao X, Moya B, Jiao Y, Basso KB, Zhou J, Lang Y, Sutaria DS, Zavascki AP, Barth AL, Reeve SM, Schweizer HP, Deveson Lucas D, Boyce JD, Bonomo RA, Lee RE, Shin BS, Louie A, Drusano GL, Bulitta JB. 2020. Novel cassette assay to quantify the outer membrane permeability of five β-Lactams simultaneously in carbapenem-resistant *Klebsiella pneumoniae* and *Enterobacter cloacae*. mBio 11:e03189-19. doi:10.1128/mBio.03189-19 PMC7018653 32047131

[48] Lakaye B, Dubus A, Joris B, Frère JM. 2002. Method for estimation of low outer membrane permeability to beta-lactam antibiotics. Antimicrob Agents Chemother 46:2901–2907. doi:10.1128/AAC.46.9.2901-2907.2002 PMC127435 12183245

[49] Asín-Prieto E, Rodríguez-Gascón A, Trocóniz IF, Soraluce A, Maynar J, Sánchez-Izquierdo JÁ, Isla A. 2014. Population pharmacokinetics of piperacillin and tazobactam in critically ill patients undergoing continuous renal replacement therapy: application to pharmacokinetic/pharmacodynamic analysis. J Antimicrob Chemother 69:180–189. doi:10.1093/jac/dkt304 23908259

[50] Lenhard JR, Bulman ZP. 2019. Inoculum effect of β-lactam antibiotics. J Antimicrob Chemother 74:2825–2843. doi:10.1093/jac/dkz226 PMC6753498 31170287

[51] Elmokadem A, Riggs MM, Baron KT. 2019. Quantitative systems pharmacology and physiologically-based pharmacokinetic modeling with mrgsolve: a hands-on tutorial. CPT Pharmacometrics Syst Pharmacol 8:883–893. doi:10.1002/psp4.12467 PMC6930861 31652028

[52] Li H, Durbin R. 2010. Fast and accurate long-read alignment with Burrows-Wheeler transform. Bioinformatics 26:589–595. doi:10.1093/bioinformatics/btp698 PMC2828108 20080505

[53] Kearse M, Moir R, Wilson A, Stones-Havas S, Cheung M, Sturrock S, Buxton S, Cooper A, Markowitz S, Duran C, Thierer T, Ashton B, Meintjes P, Drummond A. 2012. Geneious basic: an integrated and extendable desktop software platform for the organization and analysis of sequence data. Bioinformatics 28:1647–1649. doi:10.1093/bioinformatics/bts199 PMC3371832 22543367

[54] Liu Q, Yin X, Languino LR, Altieri DC. 2018. Evaluation of drug combination effect using a bliss independence dose-response surface model. Stat Biopharm Res 10:112–122. doi:10.1080/19466315.2018.1437071 PMC6415926 30881603

[55] Smith NM, Nguyen TD, Chin WH, Sanborn JT, de Souza H, Ho BM, Luong T, Roach DR. 2023. A mechanism-based pathway toward administering highly active N-phage cocktails. Front Microbiol 14. doi:10.3389/fmicb.2023.1292618 PMC10690594 38045026

[56] Davis CM, McCutcheon JG, Dennis JJ. 2021. Aztreonam lysine increases the activity of phages E79 and phiKZ against *Pseudomonas aeruginosa* PA01. Microorganisms 9:152. doi:10.3390/microorganisms9010152 PMC7827458 33445453

[57] Burrows LL, Chow D, Lam JS. 1997. *Pseudomonas aeruginosa* B-band O-antigen chain length is modulated by Wzz (Ro1). J Bacteriol 179:1482–1489. doi:10.1128/jb.179.5.1482-1489.1997 PMC178856 9045803

[58] Hao Y, Murphy K, Lo RY, Khursigara CM, Lam JS. 2015. Single-nucleotide polymorphisms found in the *migA* and *wbpX* glycosyltransferase genes account for the intrinsic lipopolysaccharide defects exhibited by *Pseudomonas aeruginosa* PA14 . J Bacteriol 197:2780–2791. doi:10.1128/JB.00337-15 PMC4524037 26078447

[59] Poon KKH, Westman EL, Vinogradov E, Jin S, Lam JS. 2008. Functional characterization of MigA and WapR: putative rhamnosyltransferases involved in outer core oligosaccharide biosynthesis of *Pseudomonas aeruginosa*. J Bacteriol 190:1857–1865. doi:10.1128/JB.01546-07 PMC2258888 18178733

[60] Bulssico J, Panigrahi S, Matveeva M, Ginet N, Ansaldi M. 2025. Antibiotic-induced morphological changes enhance phage predation. PLoS Pathog 21:e1013546. doi:10.1371/journal.ppat.1013546 PMC12510666 41042833

[61] Supina BSI, Dennis JJ. 2025. The current landscape of phage-antibiotic synergistic (PAS) interactions. Antibiotics (Basel) 14:545. doi:10.3390/antibiotics14060545 PMC12190067 40558135

[62] Heilmann S, Sneppen K, Krishna S. 2012. Coexistence of phage and bacteria on the boundary of self-organized refuges. Proc Natl Acad Sci USA 109:12828–12833. doi:10.1073/pnas.1200771109 PMC3411971 22807479

[63] Wang X, Nong L, Schaar G, Koenders B, Jonker M, de Leeuw W, ter Kuile BH. 2025. Collateral sensitivity and cross-resistance in six species of bacteria exposed to six classes of antibiotics. Microbiol Spectr 13:e00983–25. doi:10.1128/spectrum.00983-25 PMC12323328 40539805

[64] Pál C, Papp B, Lázár V. 2015. Collateral sensitivity of antibiotic-resistant microbes. Trends Microbiol 23:401–407. doi:10.1016/j.tim.2015.02.009 PMC5958998 25818802

[65] Allen RC, Pfrunder-Cardozo KR, Hall AR. 2021. Collateral sensitivity interactions between antibiotics depend on local abiotic conditions. mSystems 6:e0105521. doi:10.1128/mSystems.01055-21 PMC8631318 34846167

[66] Chegini Z, Khoshbayan A, Taati Moghadam M, Farahani I, Jazireian P, Shariati A. 2020. Bacteriophage therapy against *Pseudomonas aeruginosa* biofilms: a review. Ann Clin Microbiol Antimicrob 19:45. doi:10.1186/s12941-020-00389-5 PMC7528332 32998720

[67] Kintz E, Scarff JM, DiGiandomenico A, Goldberg JB. 2008. Lipopolysaccharide o-antigen chain length regulation in *Pseudomonas aeruginosa* serogroup O11 strain PA103 . J Bacteriol 190:2709–2716. doi:10.1128/JB.01646-07 PMC2293223 18065548

