# Supplementary material for: Phage-first treatment results in subpopulation-driven collateral sensitivity to beta-lactams in an extensively drug-resistant Pseudomonas aeruginosa
Source: Antimicrob Agents Chemother. 2026 Sep 3;70(10):e00048-26. doi: 10.1128/aac.00048-26 (PMC13689456; doi:10.1128/aac.00048-26)
Supplement: Supplementary file 1 [file aac.00048-26-s0001.pdf]

# SUPPLEMENTAL MATERIALS

# Phage-first Treatment Results in Subpopulation-driven Collateral Sensitivity to Beta-lactams in an Extensively Drug-resistant *Pseudomonas aeruginosa*

**Claire Han*[,1], Karishma Patel*[,1], Haoran Gao[1], Jacob T. Sanborn[1], Thomas D. Nguyen[1], Jack F. Klem, Brian M. Ho[1], Patrick D. Kenney[2], Yanan Zhao[1], Troy D. Wood[3], Dwayne R. Roach[4], Liang Chen[1], Nicholas M. Smith[1, †]**

[1]Division of Clinical and Translational Therapeutics, School of Pharmacy and Pharmaceutical Sciences, University at Buffalo, Buffalo, New York; [2]Department of Infectious Diseases, Jacobs School of Medicine and Biomedical Sciences, University at Buffalo, Buffalo, New York; [3]Department of Chemistry, College of Arts and Sciences, University at Buffalo, Buffalo, New York; [4]Department of Biology, San Diego State University, San Diego, California

***Contributed Equally**

**†Correspondence:**
**Nicholas M. Smith, PharmD, PhD**
308 Farber Hall
Department of Pharmacy Practice
School of Pharmacy and Pharmaceutical Sciences
University at Buffalo
Buffalo, NY 14214
Email: nmsmith2@buffalo.edu

**Acknowledgements**

This work was supported by the National Institute of Allergy and Infectious Diseases of the National Institutes of Health under Award Numbers R01AI177997, R01AI165997, and R01AI148560; the National Heart, Lung, and Blood Institute of the National Institutes of Health under Award Number R01HL167828; and the National Institute on Aging of the National Institutes of Health under Award Number R01AG056939. The content is solely the responsibility of the authors and does not necessarily represent the official views of the National Institutes of Health.

**Running Title:** Phage-induced Collateral Sensitivity

**Keywords:** KPC, Pseudomonas aeruginosa, bacteriophage, beta-lactam, carbapenem, combination therapy, LPS, permeability, pharmacodynamics

**Figure S1: Schematic of the HFIM system.** Growth media is delivered via peristaltic pump into the central reservoir, where it mixes with the central compartment volume. A duet pump circulates fluid between the central reservoir and the hollow-fiber cartridge, allowing bidirectional exchange across the semi-permeable hollow fibers while retaining bacteria within the extracapillary space (cross-section, top right), where bacteria reside extracapillary to the fibers and are exposed to diffusible drug, phage, and nutrients. Antibiotics and/or phage are administered via syringe pump into the central reservoir circuit. A second peristaltic pump removes fluid from the system into a waste reservoir to maintain a constant system volume and simulate drug/phage elimination. Samples for bacterial and phage quantification are collected from the extracapillary space (cartridge) and the central reservoir to characterize pharmacodynamic and pharmacokinetic profiles, respectively.

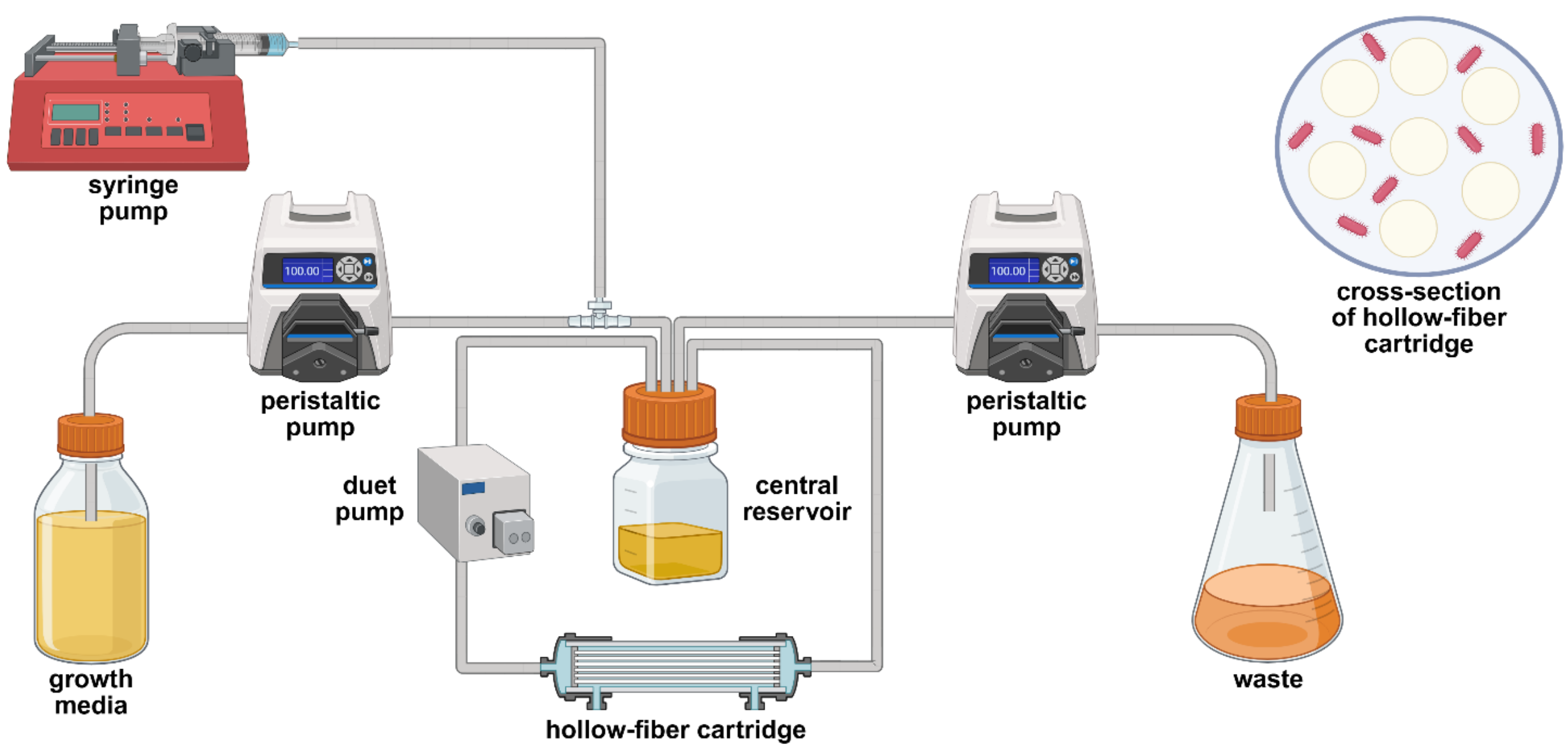

**Figure S2: Time-kill experiment used to generate PYO2-resistant mutants with AR-0231.** (A, C) Bacterial counts ($log_{10}$ CFU/ml) and (B, D) corresponding PYO2 phage concentrations ($log_{10}$ PFU/ml) over 24 hours. X-axis represents time in hours.

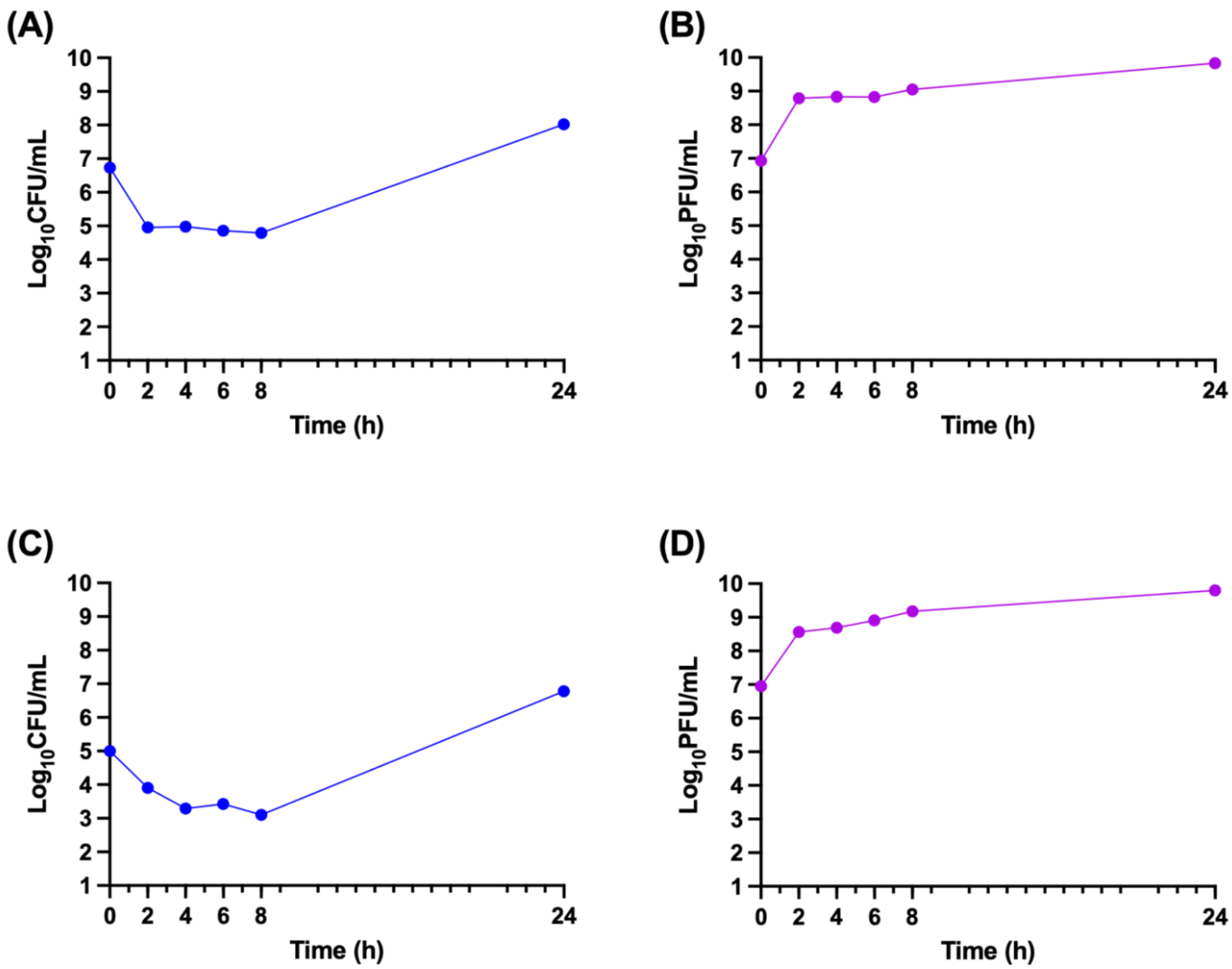

**Figure S3: Bliss Independence Analysis.** Forest plot of excess-over-Bliss values with 95% confidence intervals for ATM/AVI + PYO2 and PMB + PYO2 combinations against AR-0231 across tested antibiotic concentrations and PYO2 titers, with asterisks denoting statistical significance versus additivity (EOB = 0). Scatter plot of observed versus expected (Bliss) fractional bacterial killing, where points above the line of identity indicate synergy. Bar chart of additional $\log_{10}$ CFU/mL killing achieved beyond Bliss-predicted additivity for each antibiotic concentration and PYO2 titer combination. Error bars represent SEM; *p<0.05, **p<0.01, ***p<0.001.

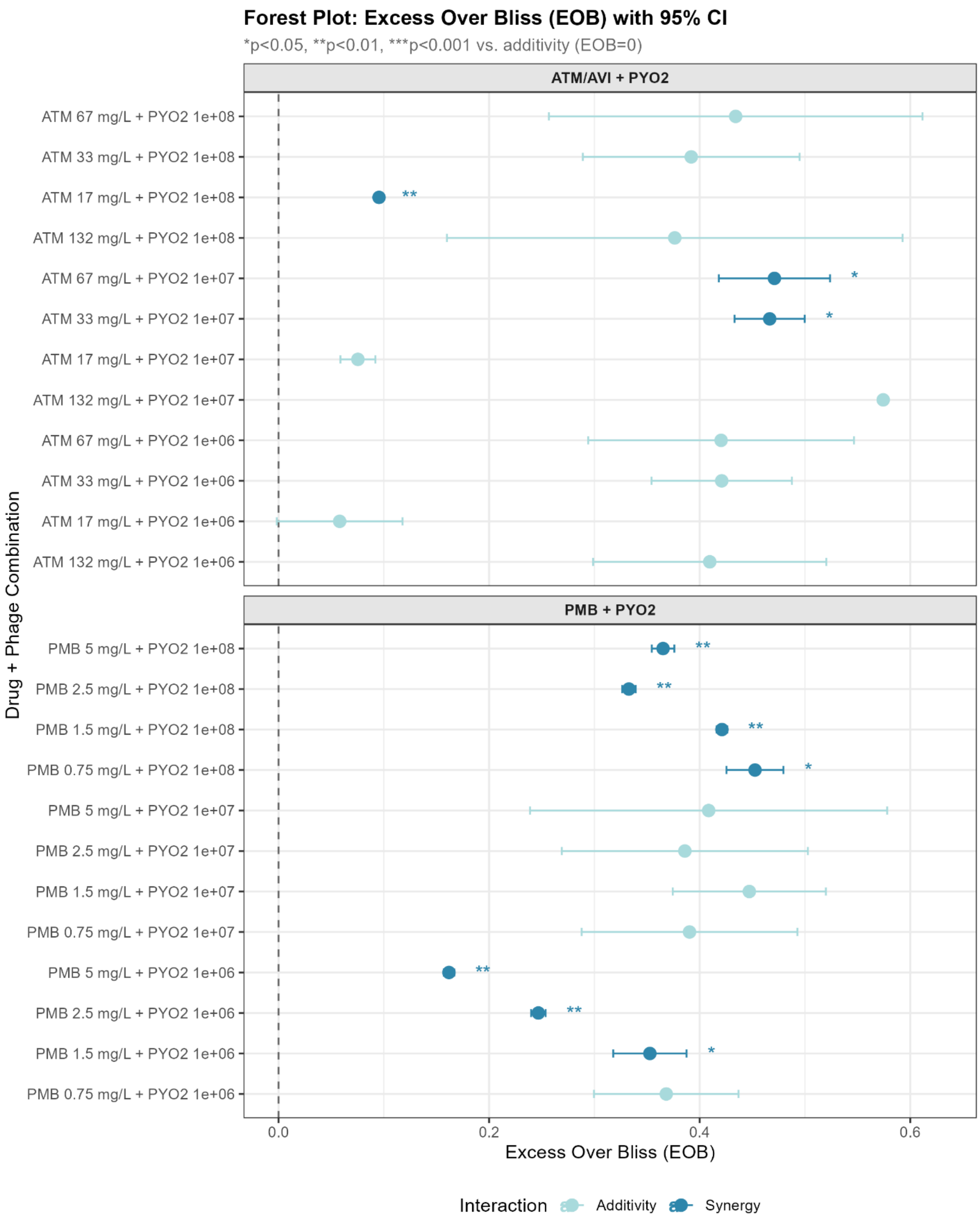

Forest Plot: Excess Over Bliss (EOB) with 95% CI
*p<0.05, **p<0.01, ***p<0.001 vs. additivity (EOB=0)
ATM/AVI + PYO2
ATM 67 mg/L + PYO2 1e+08
ATM 33 mg/L + PYO2 1e+08
ATM 17 mg/L + PYO2 1e+08
ATM 132 mg/L + PYO2 1e+08
ATM 67 mg/L + PYO2 1e+07
ATM 33 mg/L + PYO2 1e+07
ATM 17 mg/L + PYO2 1e+07
ATM 132 mg/L + PYO2 1e+07
ATM 67 mg/L + PYO2 1e+06
ATM 33 mg/L + PYO2 1e+06
ATM 17 mg/L + PYO2 1e+06
ATM 132 mg/L + PYO2 1e+06
PMB + PYO2
PMB 5 mg/L + PYO2 1e+08
PMB 2.5 mg/L + PYO2 1e+08
PMB 1.5 mg/L + PYO2 1e+08
PMB 0.75 mg/L + PYO2 1e+08
PMB 5 mg/L + PYO2 1e+07
PMB 2.5 mg/L + PYO2 1e+07
PMB 1.5 mg/L + PYO2 1e+07
PMB 0.75 mg/L + PYO2 1e+07
PMB 5 mg/L + PYO2 1e+06
PMB 2.5 mg/L + PYO2 1e+06
PMB 1.5 mg/L + PYO2 1e+06
PMB 0.75 mg/L + PYO2 1e+06
Drug + Phage Combination
0.0
0.2
0.4
0.6
Excess Over Bliss (EOB)
Interaction
Additivity
Synergy

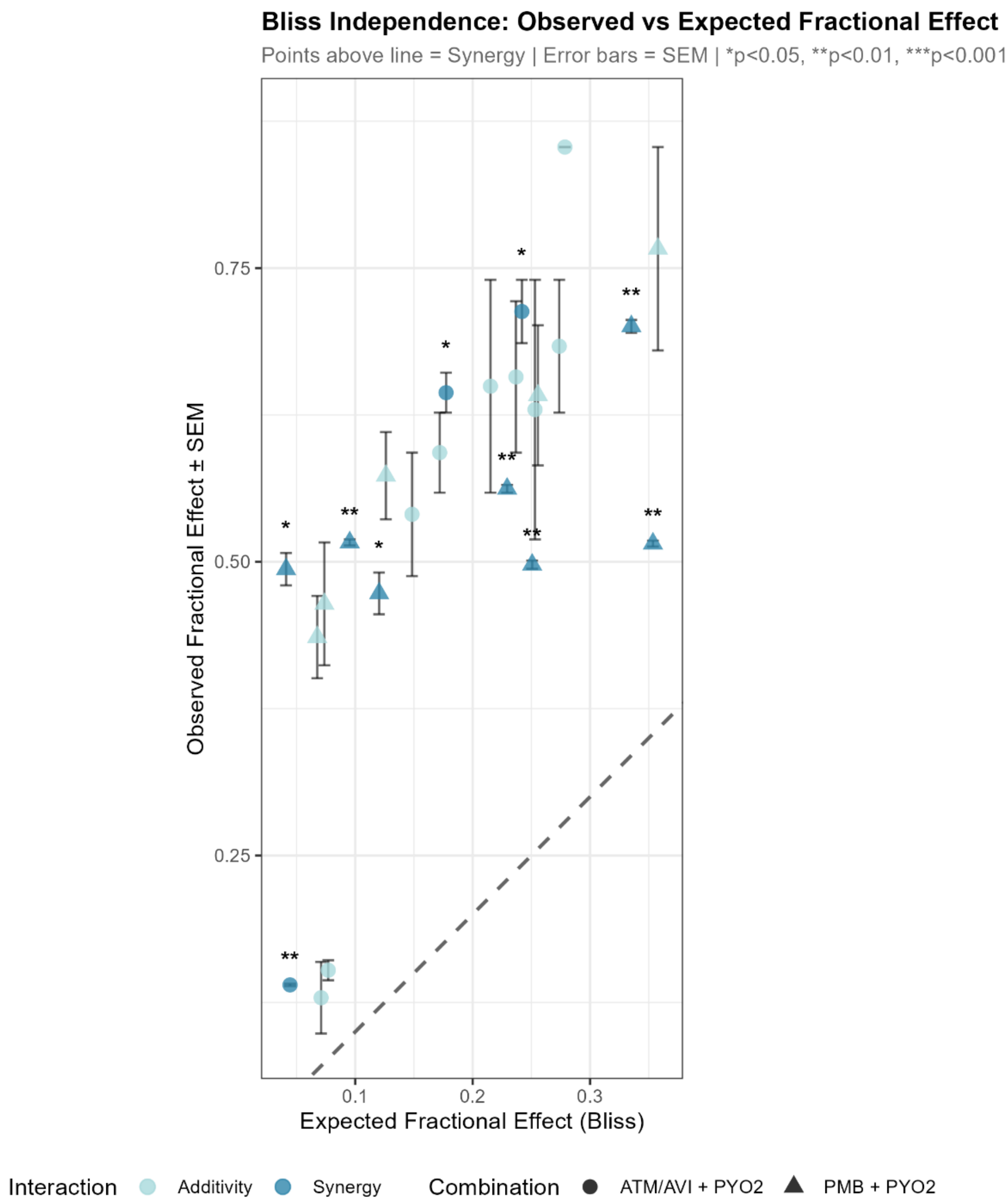

Bliss Independence: Observed vs Expected Fractional Effect
Points above line = Synergy | Error bars = SEM | *p<0.05, **p<0.01, ***p<0.001
Observed Fractional Effect ± SEM
0.75
0.50
0.25
0.1
0.2
0.3
Expected Fractional Effect (Bliss)
Interaction
Additivity
Synergy
Combination
ATM/AVI + PYO2
PMB + PYO2

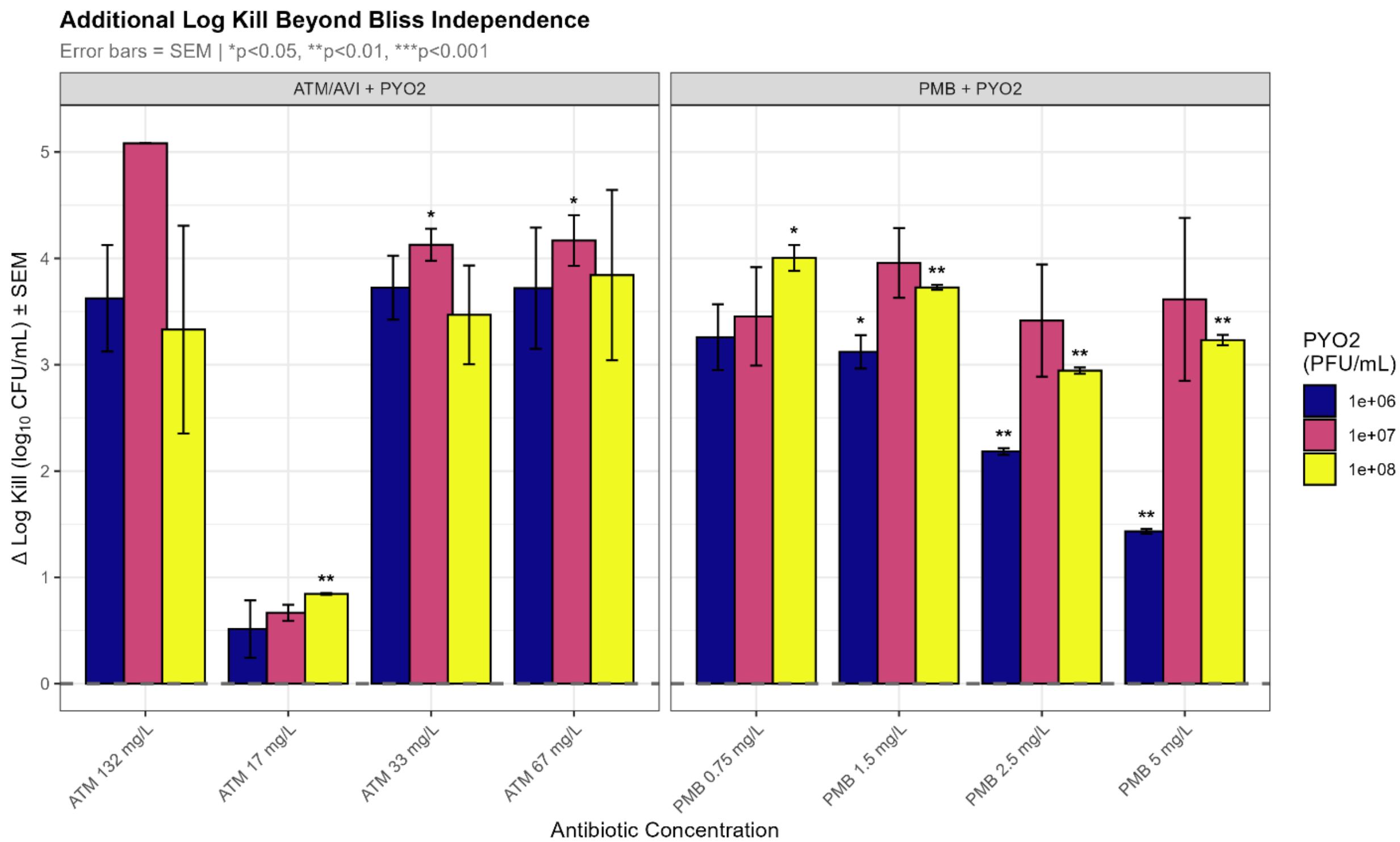


--- ATM + PYO2: Overall One-Sample t-test (H0: EOB = 0) ---

Number of observations: 24

Mean EOB: 0.3495

SD EOB: 0.1771

t-statistic: 9.6675

df: 23

p-value: 1.441e-09

95% CI: 0.2747 to 0.4243

Conclusion: Significant SYNERGY ($p < 0.05$)

--- PMB + PYO2: Overall One-Sample t-test (H0: EOB = 0) ---

Number of observations: 24

Mean EOB: 0.3611

SD EOB: 0.0903

t-statistic: 19.5925

df: 23

p-value: 7.571e-16

95% CI: 0.323 to 0.3993

Conclusion: Significant SYNERGY (p < 0.05)

**Figure S4: Concentration-time profiles of aztreonam, ceftazidime, cefepime, meropenem, imipenem, and piperacillin at concentrations 30, 10, 3, 1, and 0.3 mg/L in AR-0231.** Intact and lysed cells are graphed separately.

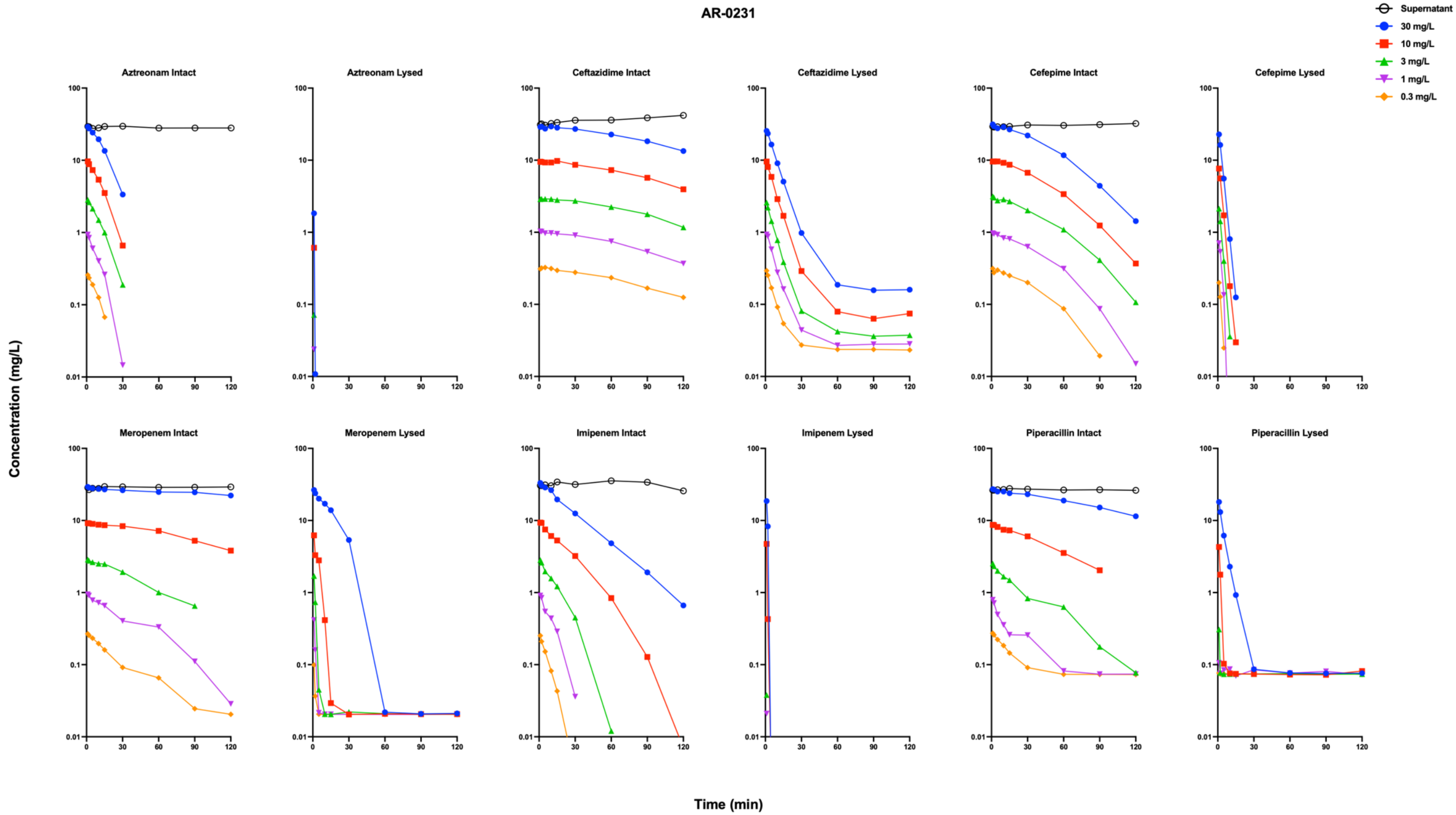

**Figure S5. Concentration-time profiles of aztreonam, ceftazidime, cefepime, meropenem, imipenem, and piperacillin: at concentrations 30, 10, 3, 1, and 0.3 mg/L in PYO2-resistant AR-0231.** Intact and lysed cells are graphed separately.

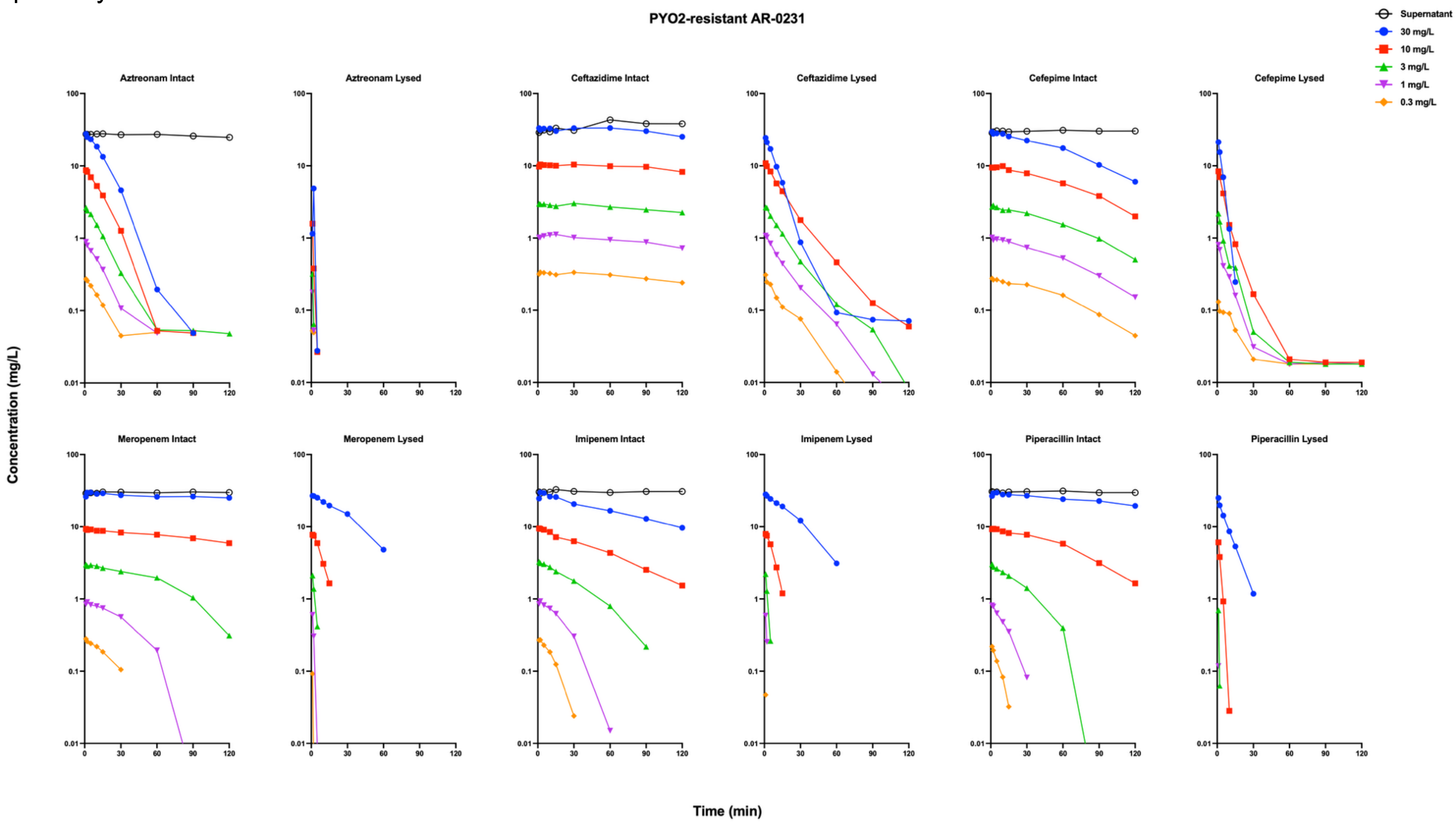

## Supplemental Mathematical Modeling Methodology

A minimal thermal degradation of the beta-lactams was shown in the control matrix over the 120-minute study period, corresponding to a previous comprehensive stability evaluation for the beta-lactams (1). The thermal degradation term $CL_{deg}$ was fixed to 0 for all the drugs except for Ceftazidime. For Ceftazidime and Piperacillin models, scaled normalizations were made with the observations in intact and lysed cells by the control matrix to account for the bias due to the instability of the internal standard which was determined to undergo hydrogen exchange with deuterated moieties and no other commercially available isotopically labeled standards were available for replacement. A correction term $K_{corr}$ is used additional to capture the observed increasing trend in Ceftazidime concentration in the control matrix.

**Figure S6: Goodness of fit Plots for Six beta-lactams.** From left to right are-(1) Observed versus Predicted Concentration Plot, (2) Conditional Weighted Residuals Plots versus Predicted Concentration Plot and (3) Conditional Weighted Residuals Plots versus Time Plot.

The dashed line in (1) represents the line of identity and in (2) and (3) represents the line of CWRES equal to +2 or -2. The red solid line represents the loess regression.

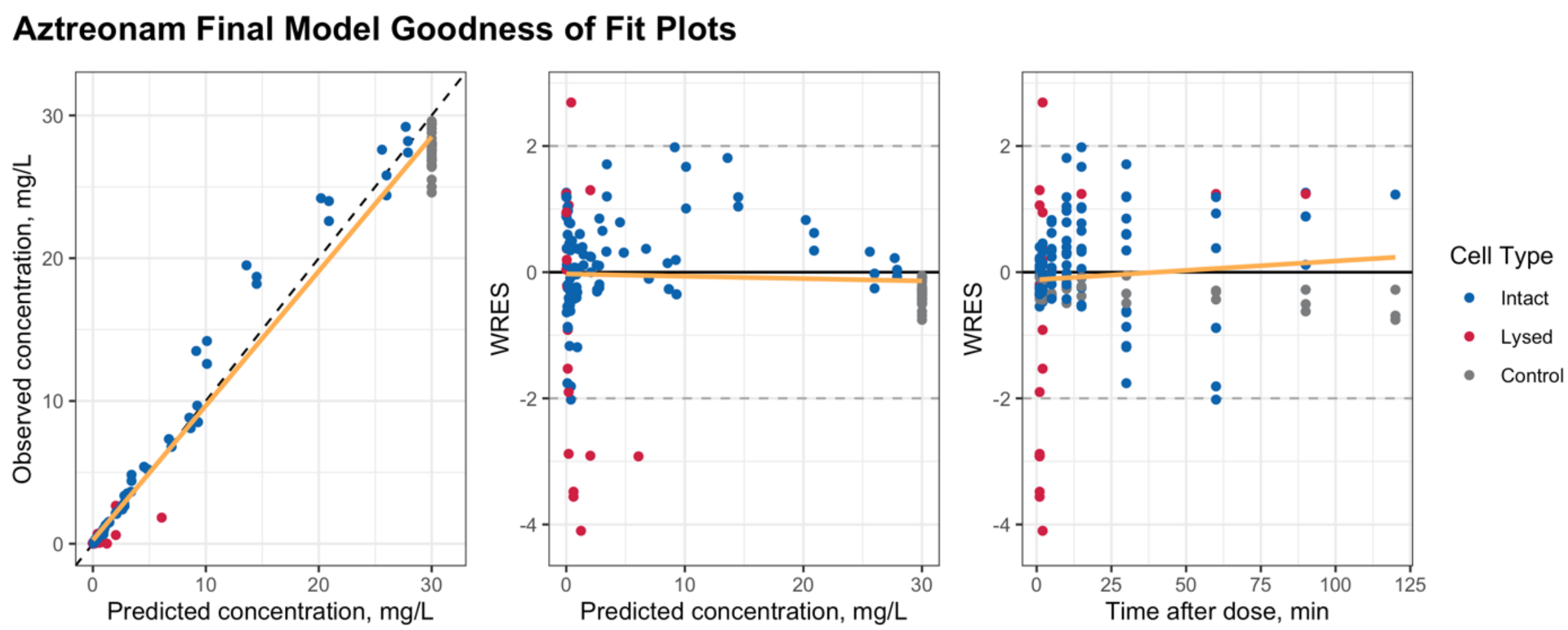


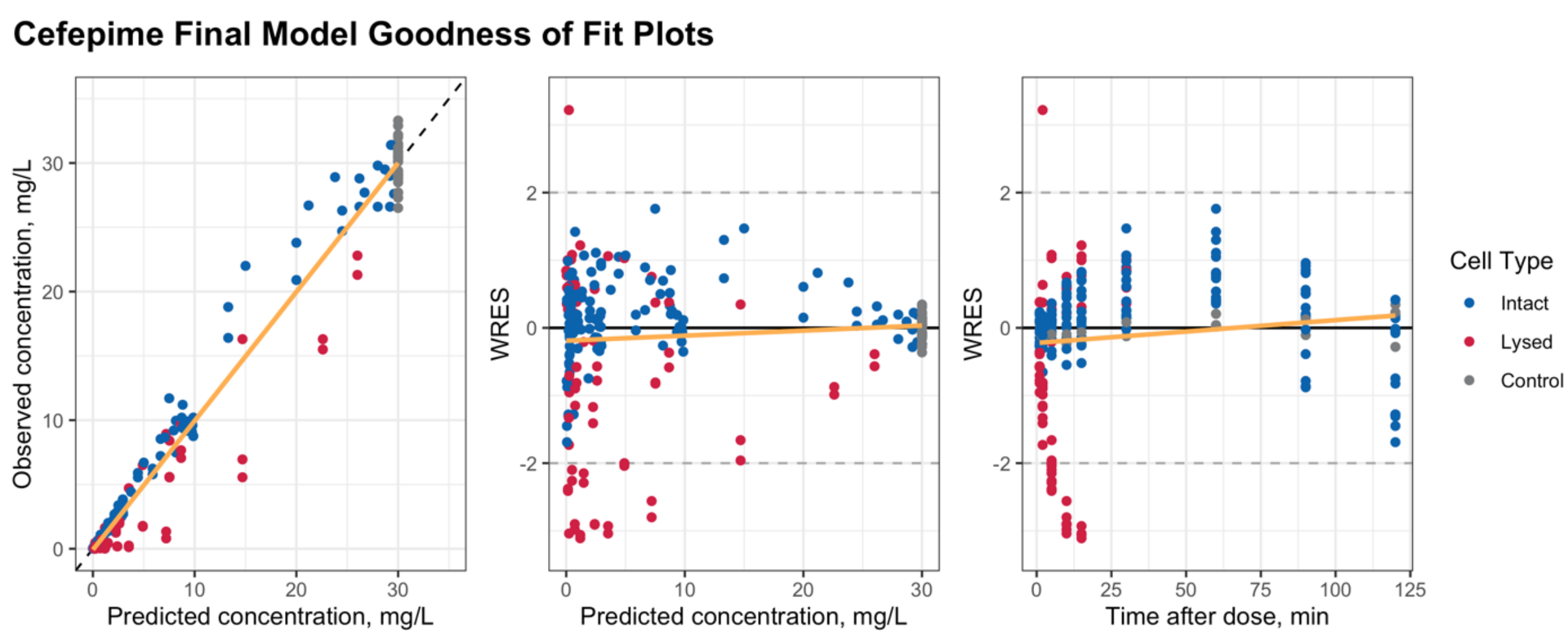

## Ceftazidime Final Model Goodness of Fit Plots

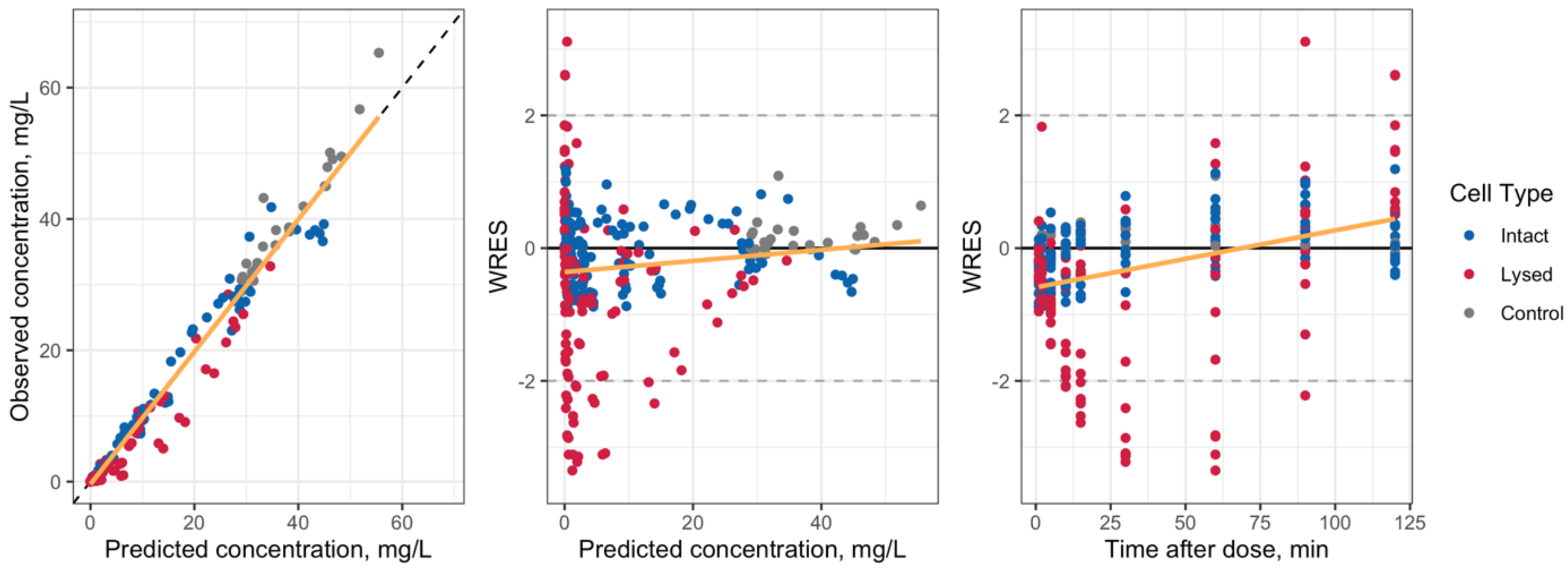


## Imipenem Final Model Goodness of Fit Plots

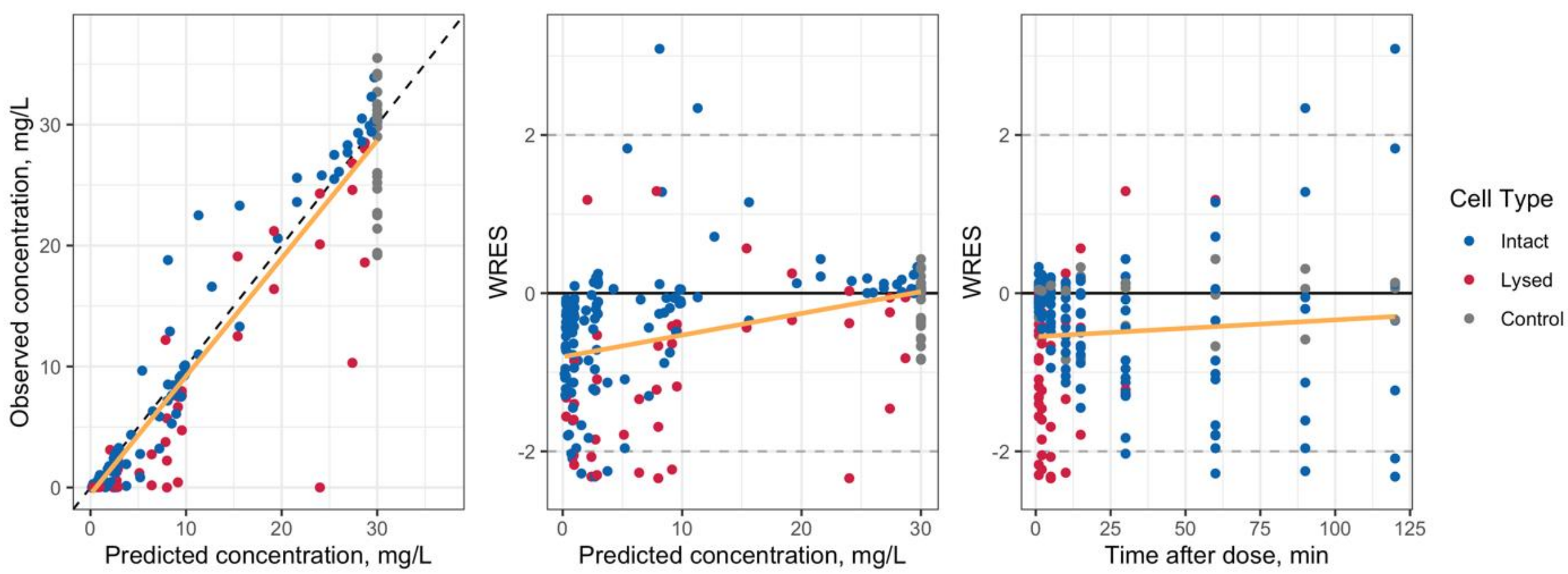

**Meropenem Final Model Goodness of Fit Plots**

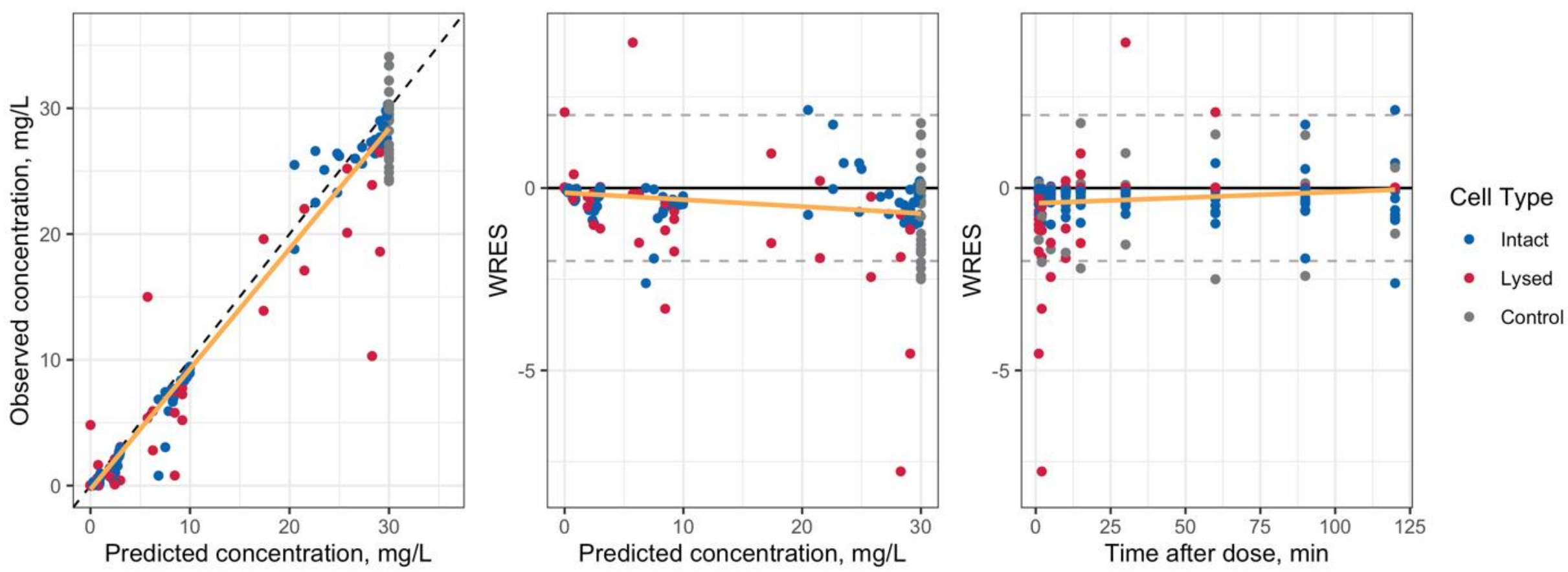


**Piperacillin Final Model Goodness of Fit Plots**

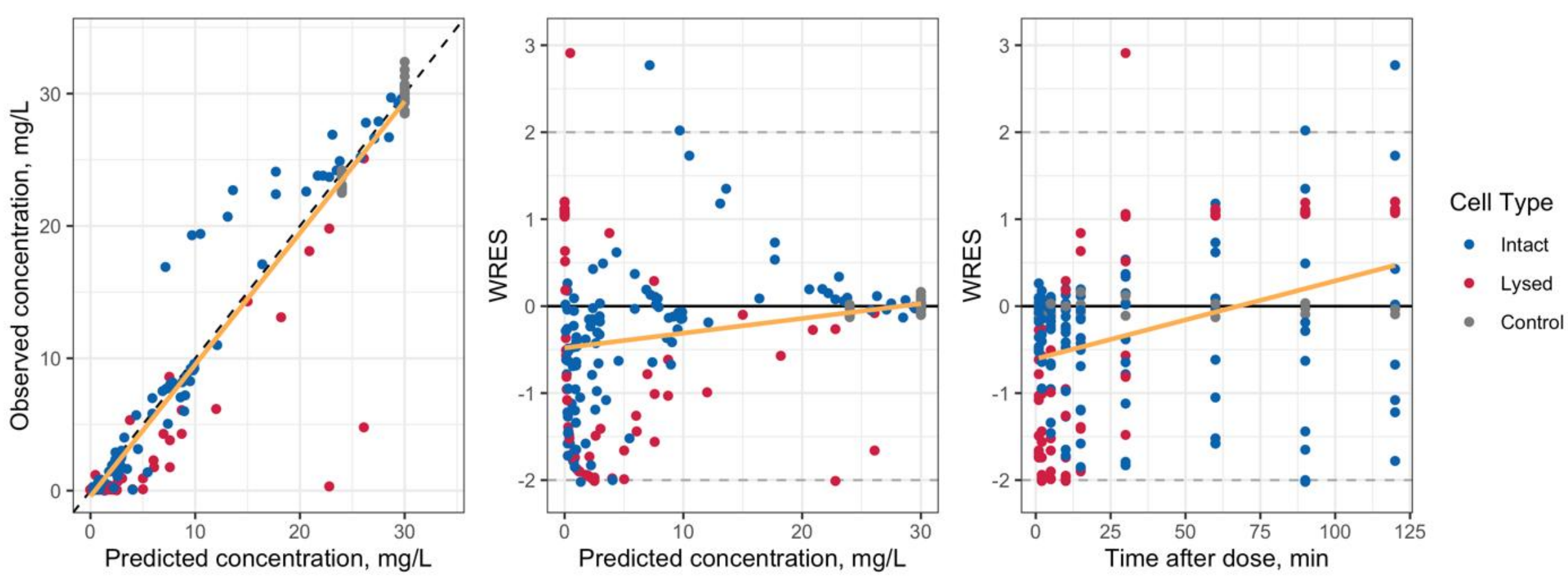

**Figure S7: Observed versus Predicted Beta-lactams Plots.** Each plot indicates observed versus predicted concentration of different beta-lactams.

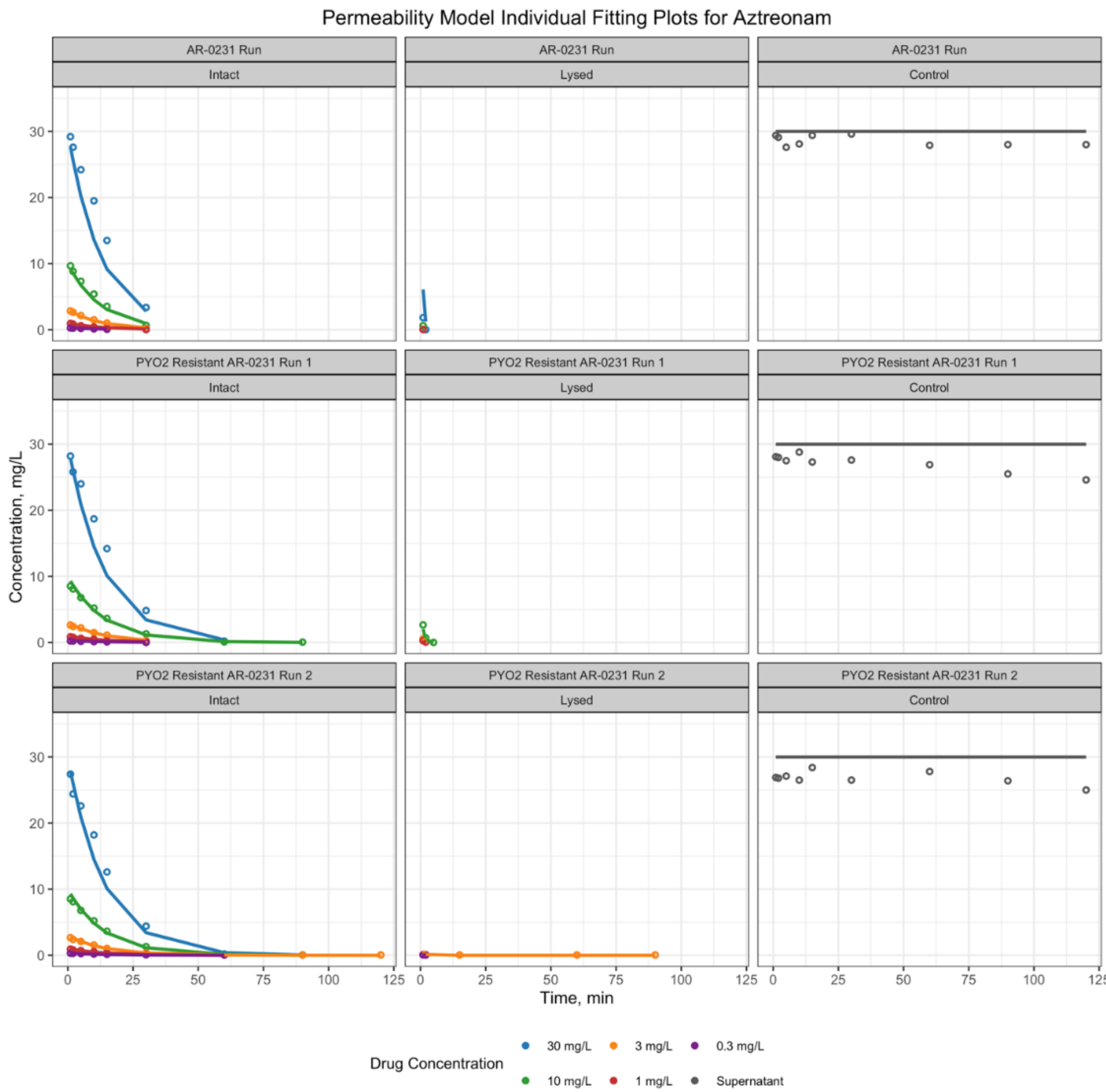

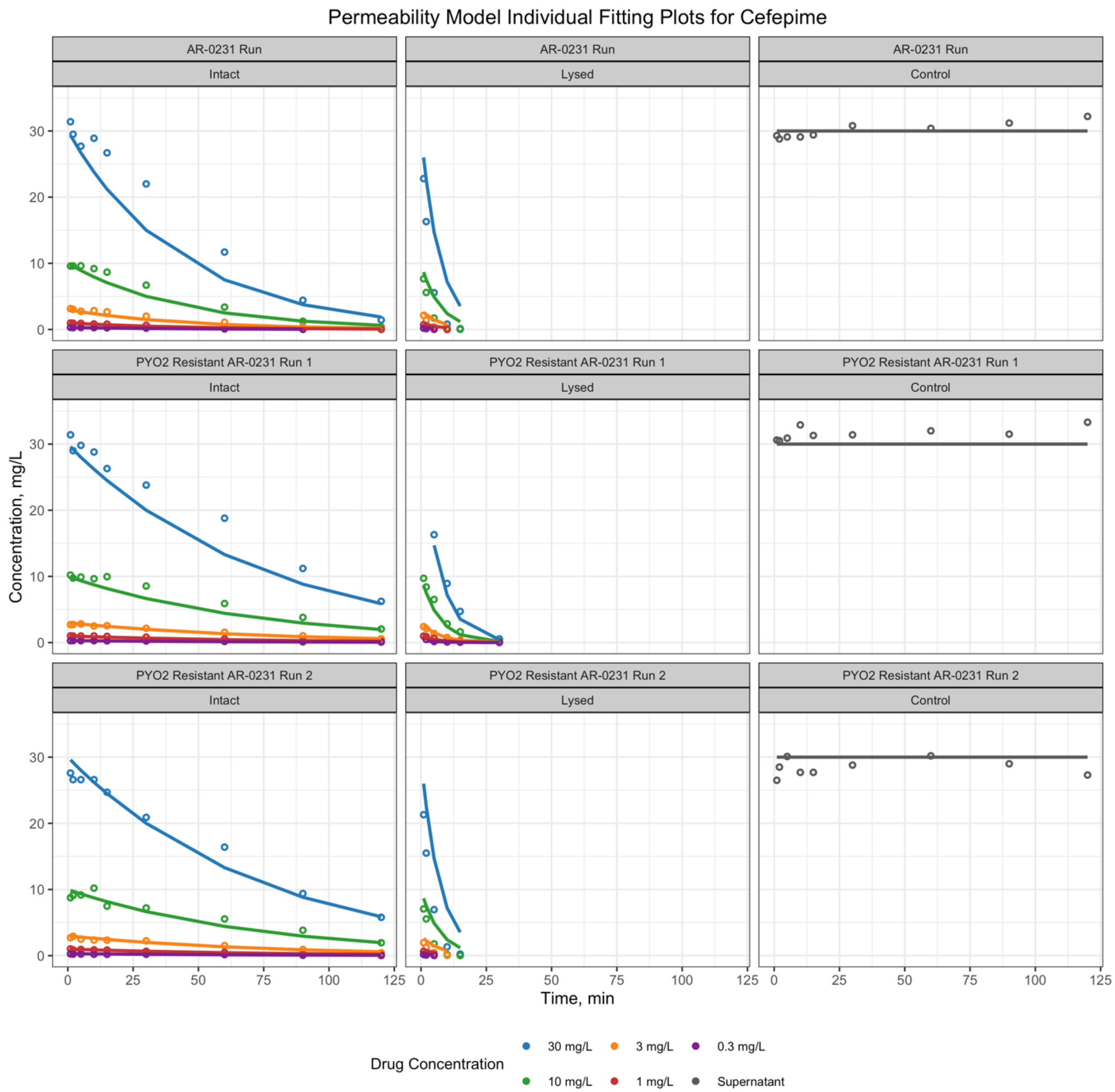

Permeability Model Individual Fitting Plots for Cefepime
AR-0231 Run
Intact
AR-0231 Run
Lysed
AR-0231 Run
Control
PYO2 Resistant AR-0231 Run 1
Intact
PYO2 Resistant AR-0231 Run 1
Lysed
PYO2 Resistant AR-0231 Run 1
Control
PYO2 Resistant AR-0231 Run 2
Intact
PYO2 Resistant AR-0231 Run 2
Lysed
PYO2 Resistant AR-0231 Run 2
Control
Concentration, mg/L
Time, min
0
10
20
30
0
25
50
75
100
125
Drug Concentration
30 mg/L
3 mg/L
0.3 mg/L
10 mg/L
1 mg/L
Supernatant

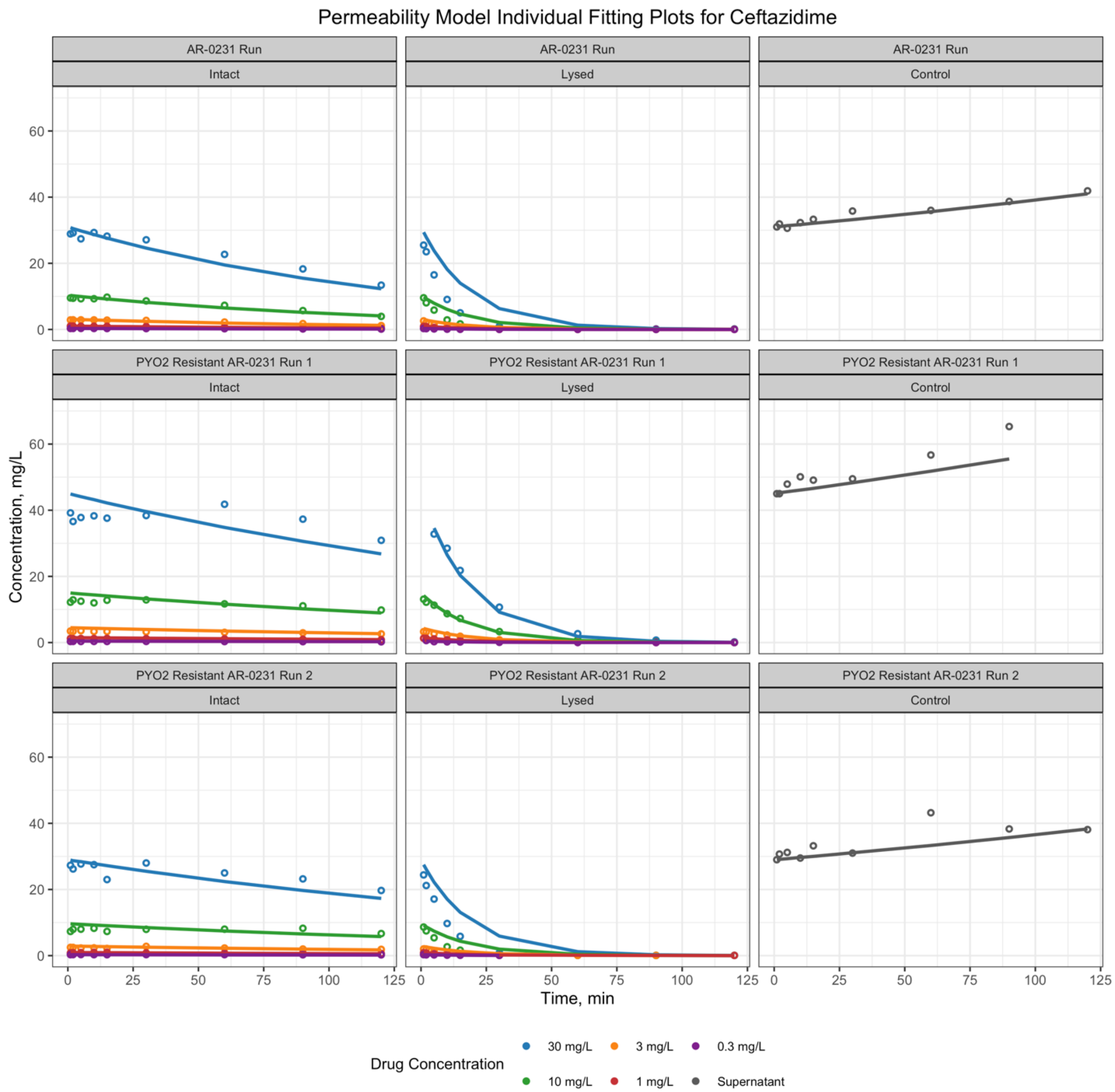

Permeability Model Individual Fitting Plots for Ceftazidime
AR-0231 Run
Intact
AR-0231 Run
Lysed
AR-0231 Run
Control
PYO2 Resistant AR-0231 Run 1
Intact
PYO2 Resistant AR-0231 Run 1
Lysed
PYO2 Resistant AR-0231 Run 1
Control
PYO2 Resistant AR-0231 Run 2
Intact
PYO2 Resistant AR-0231 Run 2
Lysed
PYO2 Resistant AR-0231 Run 2
Control
Concentration, mg/L
Time, min
0
20
40
60
0
25
50
75
100
125
Drug Concentration
30 mg/L
3 mg/L
0.3 mg/L
10 mg/L
1 mg/L
Supernatant

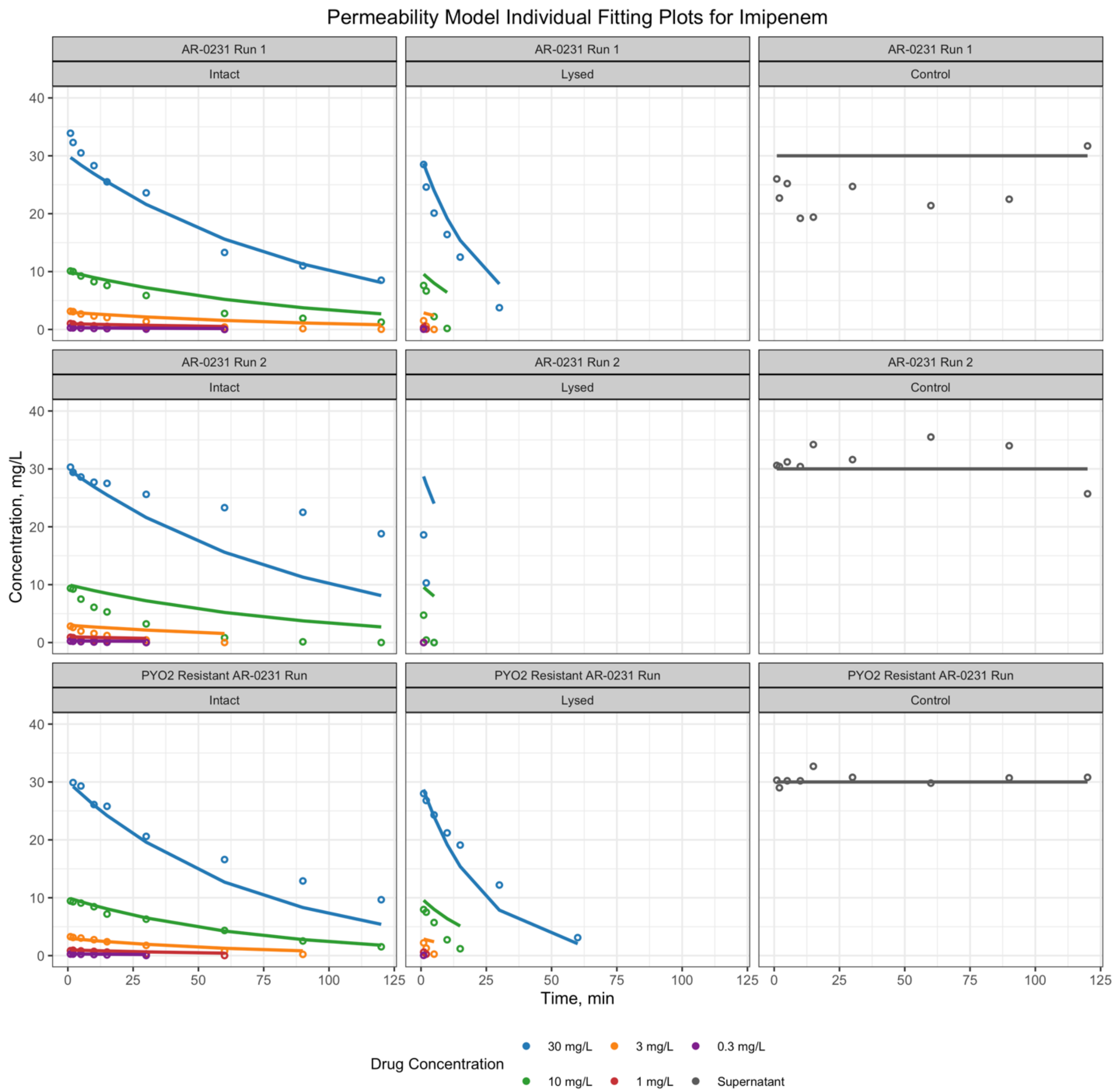

Permeability Model Individual Fitting Plots for Imipenem
AR-0231 Run 1
Intact
Lysed
Control
AR-0231 Run 2
PYO2 Resistant AR-0231 Run
Concentration, mg/L
Time, min
0
10
20
30
40
25
50
75
100
125
Drug Concentration
30 mg/L
10 mg/L
3 mg/L
1 mg/L
0.3 mg/L
Supernatant

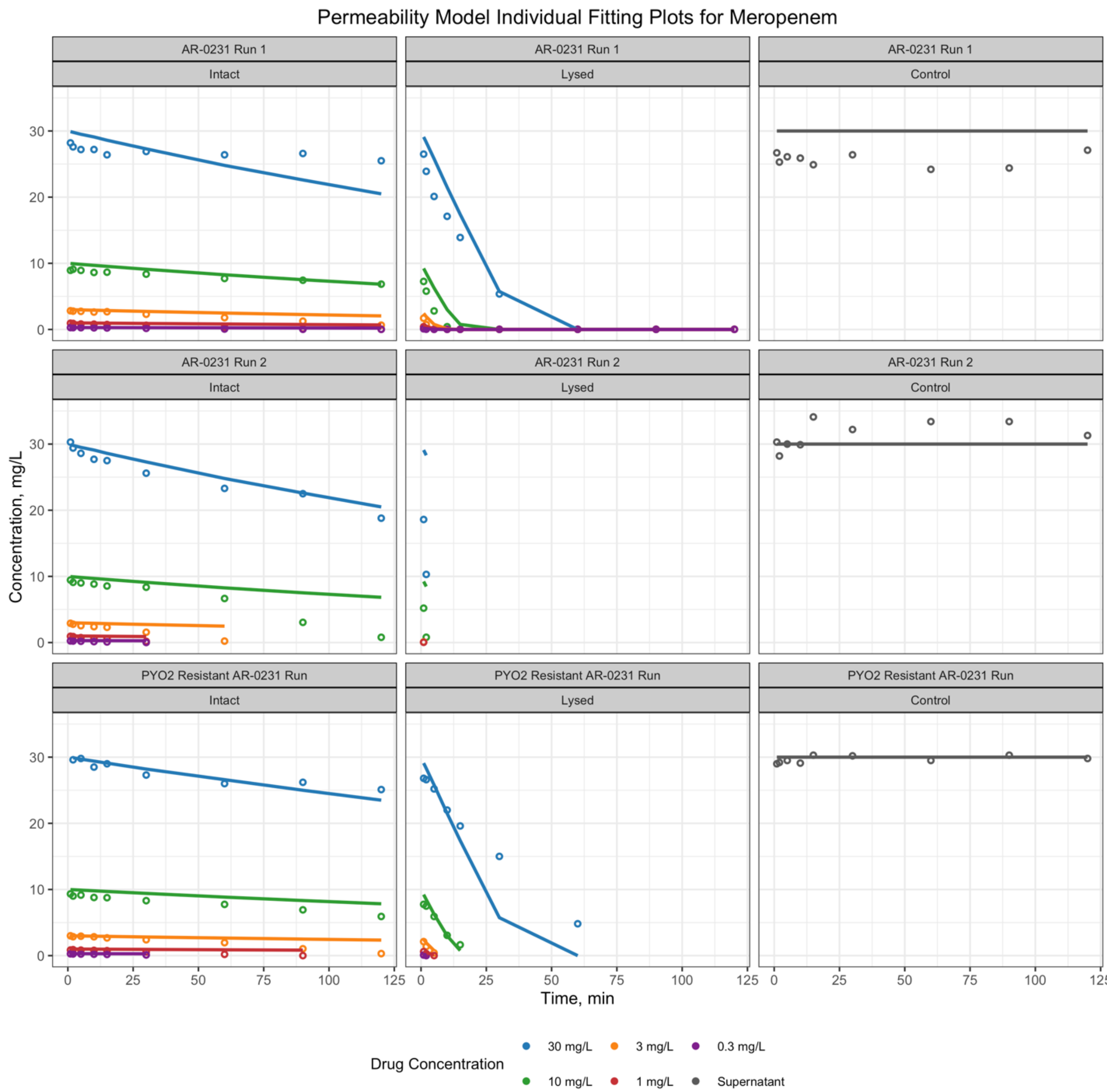
Permeability Model Individual Fitting Plots for Meropenem
AR-0231 Run 1
Intact
AR-0231 Run 1
Lysed
AR-0231 Run 1
Control
AR-0231 Run 2
Intact
AR-0231 Run 2
Lysed
AR-0231 Run 2
Control
PYO2 Resistant AR-0231 Run
Intact
PYO2 Resistant AR-0231 Run
Lysed
PYO2 Resistant AR-0231 Run
Control
Concentration, mg/L
Time, min
Drug Concentration
30 mg/L
3 mg/L
0.3 mg/L
10 mg/L
1 mg/L
Supernatant

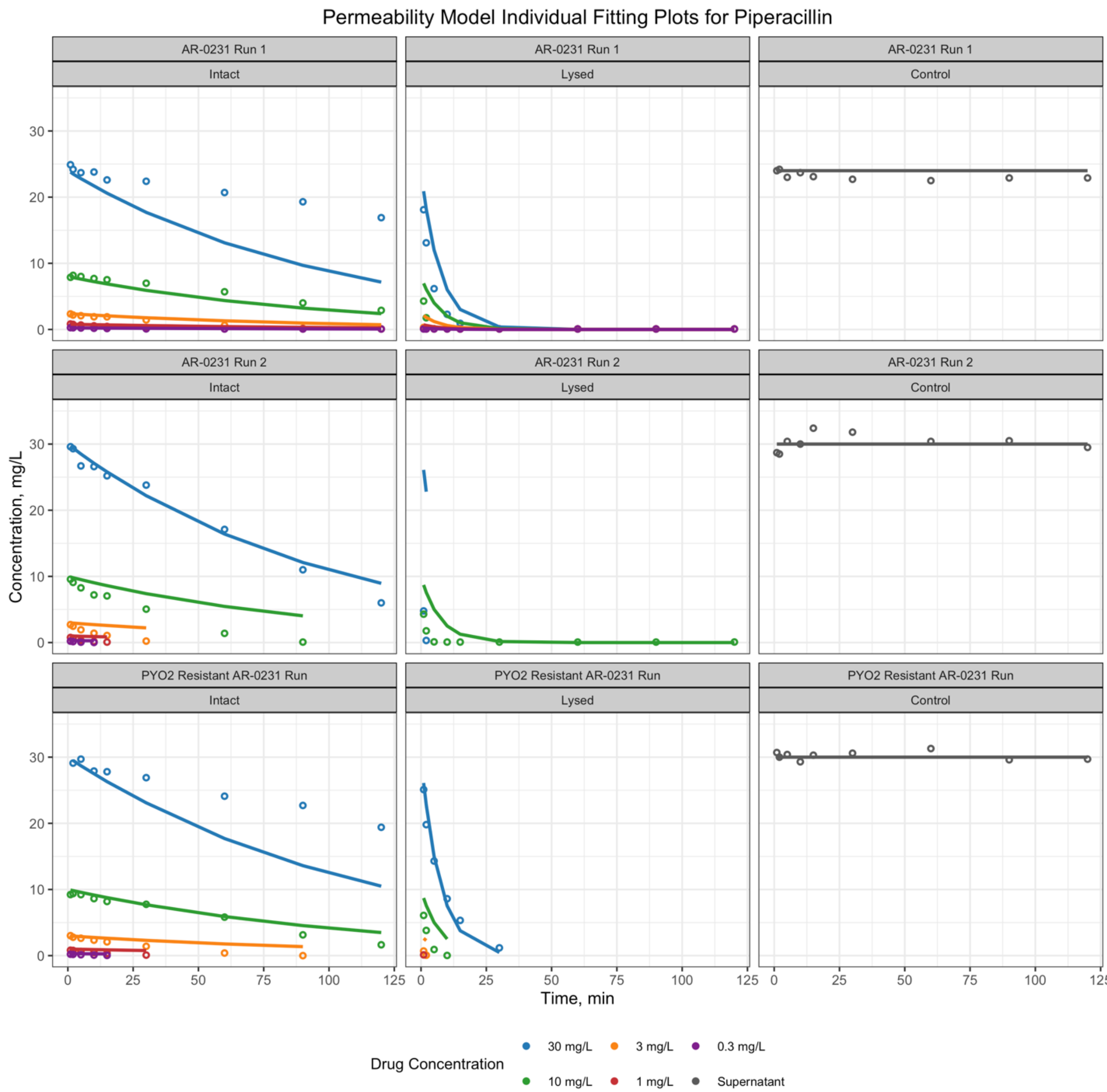
Permeability Model Individual Fitting Plots for Piperacillin
AR-0231 Run 1
Intact
Lysed
Control
AR-0231 Run 2
PYO2 Resistant AR-0231 Run
Concentration, mg/L
Time, min
0
10
20
30
0
25
50
75
100
125
Drug Concentration
30 mg/L
3 mg/L
0.3 mg/L
10 mg/L
1 mg/L
Supernatant

**Table S1: Summary of simulated unbound average steady state beta-lactams plasma concentrations.**

| Drug | Maximal daily dose in critically ill patients (g) | Unbound average steady state beta-lactamase plasma concentrations (mg/L) | Source | MW(2) |
|---|---|---|---|---|
| Aztreonam (ATM) | 8 | 22.2 | (3) | 435.433 |
| Ceftazidime (CAZ) | 6 | 24.1 | (3) | 546.576 |
| Cefepime (FEP) | 6 | 27.1 | (3) | 480.561 |
| Imipenem (IPM) | 4 | 18.9 | (3) | 299.346 |
| Meropenem (MEM) | 6 | 23.6 | (3) | 383.463 |
| Piperacillin (PIP) | 12 | 47.8 | Unpublished data | 517.555 |

**Figure S8: Simulated intracellular pharmacokinetics profiles for the AR-0231 strain and PYO2-resistant AR-0231.** Each plot indicates the predicted beta-lactam intracellular concentrations for the AR-0231 strain and the PYO2-resistant AR-0231.

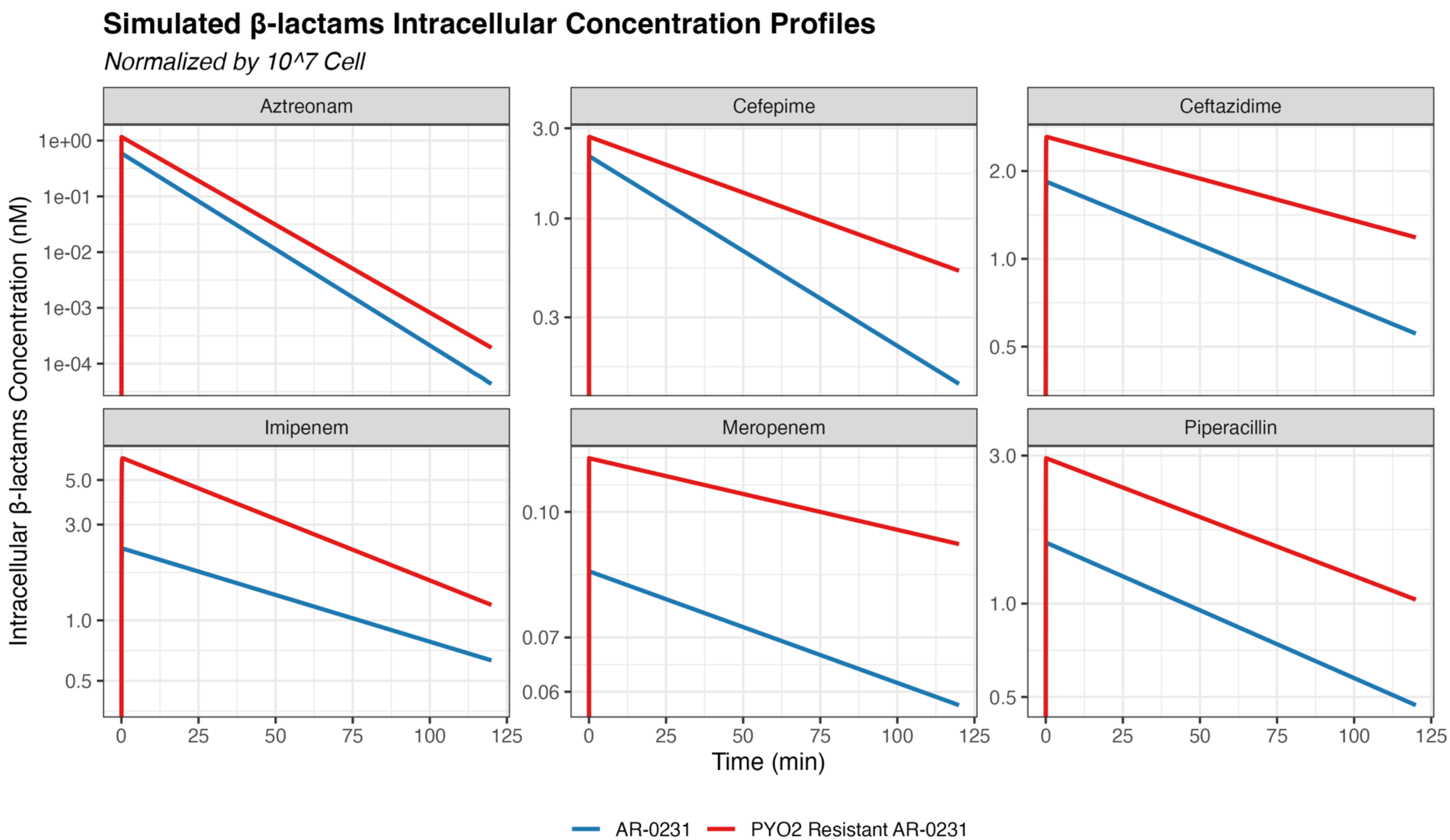

**Table S2: Minimum inhibitory concentrations (MICs) of anti-Pseudomonal beta-lactams against wild-type AR-0231 and the PYO2-resistant mutant AR0231-PYO2R.** MICs were determined for aztreonam, cefepime, ceftazidime, imipenem, meropenem, and piperacillin/tazobactam, with corresponding CLSI susceptibility interpretations for each isolate.

| | ***P. aeruginosa*** **(strain AR-0231)** | | ***P. aeruginosa*** **(strain AR0231-PYO2R)** | |
|---|---|---|---|---|
| | MIC (µg/ml) | INT | MIC (µg/ml) | INT |
| Aztreonam | > 64 | R | > 64 | R |
| Cefepime | > 32 | R | > 64 | R |
| Ceftazidime | > 64 | R | > 64 | R |
| Imipenem | > 64 | R | > 64 | R |
| Meropenem | > 8 | R | > 64 | R |
| Piperacillin/tazobactam | 128 | R | > 64 | R |

## References


1. Zhou J, Qian Y, Lang Y, Zhang Y, Tao X, Moya B, Sayed ARM, Landersdorfer CB, Shin E, Werkman C, Smith NM, Kim TH, Kumaraswamy M, Shin BS, Tsuji BT, Bonomo RA, Lee RE, Bulitta JB. 2024. Comprehensive stability analysis of 13 β-lactams and β-lactamase inhibitors in in vitro media, and novel supplement dosing strategy to mitigate thermal drug degradation. Antimicrob Agents Chemother 68:e0139923.
2. Knox C, Wilson M, Klinger CM, Franklin M, Oler E, Wilson A, Pon A, Cox J, Chin NEL, Strawbridge SA, Garcia-Patino M, Kruger R, Sivakumaran A, Sanford S, Doshi R, Khetarpal N, Fatokun O, Doucet D, Zubkowski A, Rayat DY, Jackson H, Harford K, Anjum A, Zakir M, Wang F, Tian S, Lee B, Liigand J, Peters H, Wang RQR, Nguyen T, So D, Sharp M, da Silva R, Gabriel C, Scantlebury J, Jasinski M, Ackerman D, Jewison T, Sajed T, Gautam V, Wishart DS. 2024. DrugBank 6.0: the DrugBank Knowledgebase for 2024. Nucleic Acids Res 52:D1265-d1275.
3. Smith NM, Boissonneault KR, Chen L, Petraitis V, Petraitiene R, Tao X, Zhou J, Lang Y, Kavaliauskas P, Bulman ZP, Holden PN, Cha R, Bulitta JB, Kreiswirth BN, Walsh TJ, Tsuji BT. 2022. Mechanistic Insights to Combating NDM- and CTX-M-Coproducing Klebsiella pneumoniae by Targeting Cell Wall Synthesis and Outer Membrane Integrity. Antimicrob Agents Chemother 66:e0052722.
